# Multi-target Phenylpropanoids Against Epilepsy

**DOI:** 10.2174/1570159X22666240524160126

**Published:** 2024-05-27

**Authors:** Teresa Carolliny Moreira Lustoza Rodrigues, Arthur Lins Dias, Aline Matilde Ferreira dos Santos, Alex France Messias Monteiro, Mayara Cecile Nascimento Oliveira, Hugo Fernandes Oliveira Pires, Natália Ferreira de Sousa, Mirian Graciela da Silva Stiebbe Salvadori, Marcus Tullius Scotti, Luciana Scotti

**Affiliations:** 1 Cheminformatics Laboratory, Institute of Drugs and Medicines Research, Federal University of Paraíba, 58051-900, João Pessoa, Paraíba, Brazil;; 2 Psychopharmacology Laboratory, Institute of Drugs and Medicines Research, Federal University of Paraíba, 58051-085, João Pessoa, Paraíba, Brazil;; 3 Teaching and Research Management, University Hospital Lauro Wanderley, Federal University of Paraíba, 58050-585, João Pessoa, PB, Brazil

**Keywords:** Epilepsy, phenylpropanoids, multi-target, molecular docking, GABAA, AMPA

## Abstract

Epilepsy is a neurological disease with no defined cause, characterized by recurrent epileptic seizures. These occur due to the dysregulation of excitatory and inhibitory neurotransmitters in the central nervous system (CNS). Psychopharmaceuticals have undesirable side effects; many patients require more than one pharmacotherapy to control crises. With this in mind, this work emphasizes the discovery of new substances from natural products that can combat epileptic seizures. Using *in silico* techniques, this review aims to evaluate the antiepileptic and multi-target activity of phenylpropanoid derivatives. Initially, ligand-based virtual screening models (LBVS) were performed with 468 phenylpropanoid compounds to predict biological activities. The LBVS were developed for the targets alpha-amino-3-hydroxy-5-methyl-4-isoxazolepropionic acid (AMPA), voltage-gated calcium channel T-type (CaV), gamma-aminobutyric acid A (GABAA), gamma-aminobutyric acid transporter type 1 (GAT-1), voltage-gated potassium channel of the Q family (KCNQ), voltage-gated sodium channel (NaV), and N-methyl D-aspartate (NMDA). The compounds that had good results in the LBVS were analyzed for the absorption, distribution, metabolism, excretion, and toxicity (ADMET) parameters, and later, the best molecules were evaluated in the molecular docking consensus. The TR430 compound showed the best results in pharmacokinetic parameters; its oral absorption was 99.03%, it did not violate any Lipinski rule, it showed good bioavailability, and no cytotoxicity was observed either from the molecule or from the metabolites in the evaluated parameters. TR430 was able to bind with GABAA (activation) and AMPA (inhibition) targets and demonstrated good binding energy and significant interactions with both targets. The studied compound showed to be a promising molecule with a possible multi-target activity in both fundamental pharmacological targets for the treatment of epilepsy.

## 1 INTRODUCTION

Epilepsy is a chronic, non-transmissible disease that, according to the World Health Organization (WHO), affects about 50 million people worldwide [1]. Its main characteristic is the occurrence of spontaneous, repetitive, and sustained seizures, which result in neurobiological, cognitive, and psychosocial consequences [2].

Such seizures are classified by the International League Against Epilepsy (ILAE) as “A transiently occurring an episode of signs and/or symptoms that ultimately result in abnormal or asynchronous neuronal activity in the brain” [3].

Epileptic seizures can occur in a focal or general way, involving voluntary or involuntary motor movements and even loss of consciousness [4]. Proper diagnosis and treatment are essential to ensure a better quality of life for people living with epilepsy [5]. Although there is a significant amount of antiepileptic drugs available on the market, about a third of the population lives with so-called treatment-refractory epilepsy [6]. This occurs when the response is not considered satisfactory, that is, when the drugs used are not able to prevent the severity or occurrence of seizures, or even when prevent, they have unwanted effects that end up causing damage to the patient's daily activities [7].

The search for new antiepileptic drugs that provide safer, more effective therapies and care for patients who live with treatment refractoriness is essential in the investigation for new pharmacological alternatives, substances of natural origin, such as phenylpropanoids, which, through their neuroprotective and anti-inflammatory activities seem to act in the modulation of the abnormal neural activity that occurs in epileptic seizures, making it a promising future treatment [8].

In addition, the so-called multi-target drugs, also known as multimodal, network, or multiple ligand therapy, deserve attention, the reason being that they are indicated as solutions for diseases that have complex etiologies and that are resistant to conventional treatments, as is the case of epilepsy. Such agents can modulate several targets simultaneously, resulting in improved efficacy and increased safety when compared to drugs and associations that target only a single target [9].

One way to optimize and assist in the development of new drugs is through computational studies, using an *in silico* approach, in which it is possible to select from a large sample those that present the best results, thus allowing the identification of promising molecules for other applications like the non-clinical *in vivo* studies. Such a preliminary study results in cost and time reduction, streamlining the process of selection and development of candidates for new drugs with therapeutic potential [10].

Thus, this work aimed to carry out a bibliographic review of studies on phenylpropanoids and their derivatives that present antiepileptic activity. In addition, to develop an experimental study to investigate, through *in silico* studies, the possible multi-target activity of phenylpropanoids against epilepsy.

## 2 PATHOPHYSIOLOGY OF EPILEPSY

The pathophysiology of epilepsy involves an imbalance between excitation (glutamatergic neurotransmission) and inhibition (GABAergic neurotransmission) of the central nervous system [11]. The occurrence of epileptic seizures is associated with the dysfunction of different mechanisms that result in changes in the brain, affecting the release of neurotransmitters, the properties of receptors and channels, synaptic reorganization, and astrocyte activity [12].

### 2.1 Gamma-aminobutyric Acid Receptors

Gamma-aminobutyric acid (GABA) is the main inhibitory transmitter in the brain and spinal cord, acting to prevent excessive excitation and coordinate neuronal activities. Inhibitory synaptic transmission is mediated by GABAergic and glycinergic ionotropic channels that are permeable to chloride (Cl^-^). The GABAergic receptors are GABAA, GABAA-rho (ionotropic receptors), and GABAB (metabotropic receptors) [13]. The GABAA and GABAA-rho receptors are ion channels that allow the influx of Cl^-^, causing a hyperpolarization in the neuronal membrane, making it difficult to trigger the action potential necessary for the release of neurotransmitters [14].

The GABAA receptor is a pentameric receptor with five subunits (two α, two β, and one ϒ or δ). Several drugs or agonists bind to this receptor. GABAB receptors act by activating second messengers of K^+^ channels and, consequently, hyperpolarization of the postsynaptic cell, reducing Ca^2+^ conductance [15, 16].

### 2.2 Alpha-amino-3-hydroxy-5-methyl-4-isoxazolepropio-nic acid (AMPA)

Alpha-amino-3-hydroxy-5-methyl-4-isoxazolepropionic acid-type glutamate receptors are ion channel ligands. They are expressed in neurons and glial cells, mediating much of the rapid excitatory transmission. Regarding structure, AMPA receptors have four subunits (GluA1, GluA2, GluA3, and GluA4) [17]. AMPA-type glutamate receptors are targets for seizure suppression due to their ability to modulate glutamatergic transmission [18]. Furthermore, the intrinsic excitability of the nervous system, which is controlled by the opening or blocking of ionic channels by voltage and regulated by the influx of cations into the interior of the neuron, plays a relevant role in the development of crises [19].

### 2.3 N-methyl-D-aspartate Receptor (NMDAR)

The N-methyl-D-aspartate post-synaptic glutamate receptor is related to different brain functions and is related to the physiopathogenesis of epilepsy through the production of epileptic focus that causes depolarizing changes, capable of producing epileptic discharges [20]. NMDARs are slowly activated in response to transient glutamate but show persistent activity and slow deactivation [21].

The development of new drugs that are antagonists of NMDA receptors has shown stimulating results due to their ability to attenuate epileptogenesis despite the presence of adverse effects that make clinical use difficult [22]. NMDA receptors are highly permeable to Ca^2+^ and contribute a slow component to the excitatory action potential [23].

Channels linked to NMDA receptors tend to stay open longer, allowing a high flow of calcium ions. So that the activation of the NMDA receptor can initiate Ca^2+^-dependent intracellular signaling cascades that will lead to changes in gene expression and synaptic strength, manifesting in functions related to learning and memory formation [24].

### 2.4 Voltage-gated Sodium Channels (NaV)

Voltage-gated sodium channels are transmembrane proteins that enable the influx of Na^+^ into excitable and non-excitable cells [25]. Sodium channels drive the generation and propagation of action potentials and can function in three states: resting (closed), activated (open), and inactivated (closed). NaV channels are arranged in different types of tissues and cells and are essential for nervous, muscular, and cardiac functioning [26].

The current-carrying sodium channels are heteromultimeric proteins composed of an A-subunit that forms large pores and B-subunits that form smaller pores. The A-subunit is organized into four domains (DI-DIV) [26]. So, each domain is composed of six transmembrane helices (S1-S6) that are connected to each other by intra and extracellular loops. The B-subunits have a single transmembrane domain whose particularity is a large extracellular domain [27].

Sodium channel blockers have been used as a pharmacological target to treat epilepsy since 1938. The drugs usually used to reduce epileptic seizures, such as phenytoin, carbamazepine, lamotrigine, and oxcarbazepine, cause rapid inactivation of the sodium channel, whereas lacosamide and eslicarbazepine acetate condition the state of slow inactivation [28].

### 2.5 The Voltage-dependent Calcium Channel (CaV)

The calcium ion (Ca^2+^) is a bivalent cation fundamental for the maintenance of all forms of life. Ca^2+^ performs several functions, enabling intracellular signaling, the release of neurotransmitters, regulation of secretion, contraction, enzymatic activity, phosphorylation, and dephosphorylation of proteins, among others [29-31]. The voltage-dependent calcium channel constitutes the main source of intracellular Ca^2+^ [32].

Intracellular calcium ions act as second messengers, important in several signaling pathways. The increase in the intracellular concentration of Ca^2+^ resulting from membrane depolarization or an action potential causes the activation of intracellular signaling cascades [33]. The CaV is related to the activity of neurons in different ways and is pointed out as an important factor in the development of epileptic seizures [34]. Thus, calcium signaling constitutes a promising target for the development of antiepileptic drugs. Some antiepileptic drugs have a modulating action on the intracellular Ca^2+^ influx [35, 36].

### 2.6 Voltage-dependent Potassium Channel (KCNQ)

Among the voltage-dependent potassium (K^+^) channels, KCNQ1 (also known as Kv7.1 or KvLQT1) stands out due to its multiple modulations by different stimuli [37]. This channel is present in numerous organs and performs essential functions in human physiology [38, 39]. The KCNQ genes are composed of five Kv7 subunits (Kv7.1-Kv7.5), four of which (Kv7.2-Kv7.5) are expressed in the CNS. The Kv7.2 (KCNQ2) and Kv7.3 (KCNQ3) subunits form a complex known as the M channel, a type of voltage-sensitive K^+^ channel that opens and closes slowly [40].

The channel is moderated by the lipid signaling of phosphatidylinositol 4,5-bisphosphate (PIP2), which in turn acts as a cofactor of KCNQ1 and is essential for the activation of KCNQ1 homotetramers [41]. The PIP2 acts by coupling the voltage sensor domain to the pore domain so that in the absence of PIP2, the pore does not open [42]. Mutations in the KCNQ2 and KCNQ3 genes cause early-onset epilepsy [43]. The most studied pharmacological modulator of Kv7 channels is retigabine; together with its structural analog, flupirtine, they are the only activators of voltage-gated potassium channels authorized for use in humans [44].

### 2.7 GABA Transporter Type 1 (GAT-1)

The GABA transporter type 1 (GAT-1) is the main cortical transporter of GABA and plays a key role in modulating GABA signaling. It is related to a wide range of brain functions and is involved in the pathophysiology of numerous neuropsychiatric diseases, including epilepsy [45-48].

GABA uptake by GAT-1 is inhibited by cis-3-amino cyclohexane carboxylic acid (ACHC) and, to a lesser extent, by 2,4-diaminobutyric acid [49, 50]. Thus, GAT-1 is strongly expressed in axon terminals [51, 52].

Tiagabine binds to GAT-1 with high affinity, constituting a clinically effective antiepileptic drug [53, 54]. The affinity of tiagabine for GAT-1 defines its action to regions of the CNS where the transporter plays an important role [55].

## 3 PHARMACOLOGICAL POTENTIAL OF PHENYLPROPANOIDS

Phenylpropanoids and their derivatives are an important class of secondary metabolites present in plants, mainly derived from phenylalanine and tyrosine, which originate through the shikimic acid pathway, and a large amount of these compounds are found in fruits, vegetables, cereal grains, and herbs [56]. In these plants, these compounds have important biological functions for the survival and development of the species, such as making the cell wall more resistant against pressure, oxidation resistance, efficient antifungal, and antimicrobial activity [57], in addition to performing an important function in the protection against environmental stresses such as ultraviolet radiation [58].

After the synthesis of phenylalanine *via* shikimate, a series of enzymatic reactions occur for the production of phenylpropanoids. The first is deamination *via* phenylalanine ammonia-lyase (PAL), producing cinnamic acid, which will subsequently be hydrolyzed by cinnamate-4 -hydroxylase (C4H), transforming into p-coumaric acid, this being an intermediate step, which in some species of fungi and bacteria can be skipped by the direct conversion made by PAL of phenylalanine into p-coumaric acid. From this, the enzyme 4-coumariol-CoA ligase (4CL) will catalyze the reaction for the formation of p-coumaroyl-CoA, and through the action of Catechol-O-Methyltransferase (COMT) methylation will occur, leading to the formation of 3,5-dimethoxy-4-hydroxycinnamic acid, or synaptic acid, leading to the generation of several phenylpropanoid compounds [59-61].

The phenylpropanoid derivatives can be divided into 7 distinct groups, namely flavonoids, lignans, simple phenolic acids, stilbenes, benzoic acids, coumarins, and tannins [62-65]. Among these, flavonoids, lignans, and phenolic acids are the most present in land plants [59], while coumarins are more abundant in fruits, such as strawberries, cherries, and apricots [66].

These phenylpropanoid metabolites present a phenyl group linked to a 3-C propane side chain; based on their basic structure, variations in the benzene ring and the position of the propenyl double bond can occur, which leads to a wide variety of compounds with diverse biological activities [67].

In this way, phenylpropanoid derivatives are becoming increasingly studied for the treatment of new diseases, in this sense, their antioxidant, neuroprotective, anti-tumor, anti-inflammatory, renoprotective, anti-diabetic, and antibacterial capabilities stand out.

Concerning neuroprotective antioxidative activity, it was identified in some compounds, such as ethyl ferulate, a phenylpropanoid derived from ferulic acid present mainly in rice and corn grains, in which it was identified in the *in vivo* and *in vitro* studies; ethyl ferulate was able to reduce lipid peroxidation and free radical levels [68-70].

The anti-tumor activity could be visualized in essential oils rich in phenylpronanoids, such as eugenol, methyleugenol, isoeugenol, and 1'-hydroxymethyleugenol, which were able to inhibit cell proliferation and cause cytotoxicity in tumor cells, as well as induce apoptosis cascades [71].

The anti-inflammatory effect of phenylpropanoids can be found in the hexane extract of *Croton veletinus*, by reducing the production of nitric oxide by inflammatory cells and also by leading to a decrease in the production of interleukin-1β (IL-1β), increasing the anti-inflammatory potential of these compounds [72].

The renoprotective action was reported in the compound p-coumaric acid, found mainly in some fruits and beverages such as grapes and coffee, which demonstrated the ability to prevent the development of severe tubular necrosis in the kidneys in adult Wistar rats [73].

Studies of the anti-diabetic effect showed that gallic acid and p-coumaric acid, both found in grapes, demonstrated anti-diabetic action by possible modulation of messenger Ribonucleic Acid (mRNA) expression of Peroxisome Proliferator-Activated Receptor γ (PPARγ), secretion of Tumor Necrosis Factor (TNF-α), and adipocytokines [74].

Antibacterial activity can be found in curcumin, from the extraction of *Curcuma longa*, when tested associated with traditional antibacterials in resistant strains. The extract proved to be able to reverse this resistance, with a better action profile in Gram-positive bacteria, especially against *Staphylococcus aureus*, in addition to other species, such as *Escherichia coli* and *Enterococcus faecalis*, proving to be a powerful synergistic agent in combating bacterial resistance [75].

Regarding the decrease in bacterial resistance, 1'S-1'-acetoxychavicol acetate, a phenylpropanoid isolated from the rhizome of *Alpinia galanga*, was able to inhibit the efflux pump of mycobacteria, thus being a potential resistance reduction mechanism for new antibacterial agents anti-tuberculosis [76].

The p-hydroxycinnamaldehyde, the active phenylpropanoid purified from the acetone fraction of *A. galanga*, was able to inhibit the effects of IL-1β, which causes a reduction in the release of extracellular matrix remodeling molecules, in addition to reducing the expression of catabolic genes, such as MMP-3 and MMP13. And induce the expression of anabolic genes, such as collagen, thus making it a good therapeutic candidate for the treatment of osteoarthritis [77].

Thus, all these examples suggest a great pharmacological potential of phenylpropanoids and their derivatives both as new therapeutic agents and as synergistic drugs, being important for the identification and better characterization of this pharmacological class for the development of new drugs.

## 4 DERIVATIVES OF PHENYLPROPANOIDS

### 4.1 Benzoic Acids

**Fig. (1):**
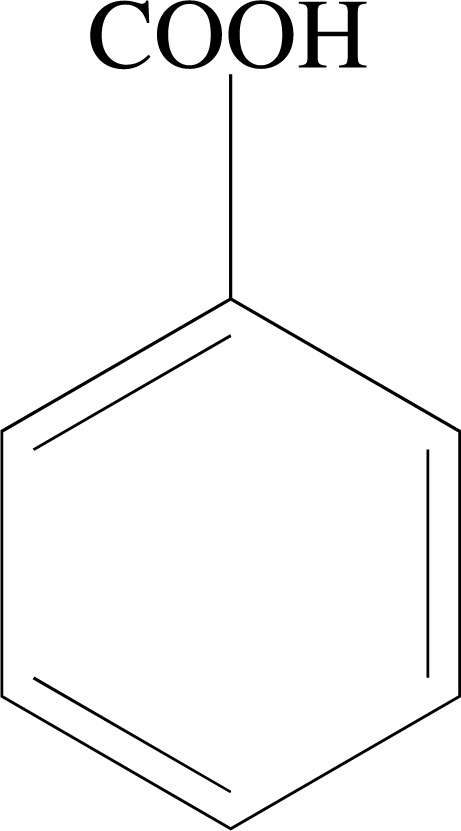
Chemical structure of Benzoic acid.

Benzoic acids (Fig. **1**) are organic acids whose carboxyl group is attached to an aromatic ring and whose biosynthesis can occur from intermediates of the shikimate or phenylpropanoid pathways [78]. With regard to metabolism, these compounds are considered extremely versatile, as they can act as precursors of primary and secondary metabolites. For example, they can produce phytohormones, attractive compounds for pollinators and seed dispersers, defense compounds, and electron transporters. In addition, some of them also have pharmacological properties or are used as raw materials for organic synthesis [78, 79].

**Fig. (2):**
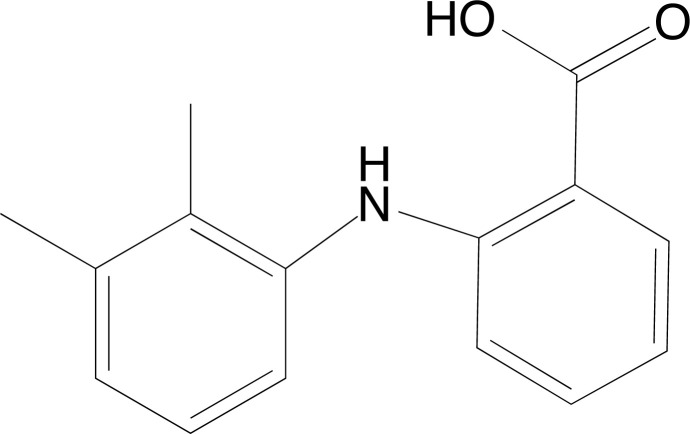
2D structure of the substance 2-[(2,3-dimethylphenyl) amino]benzoic acid.

The work by Charanya and collaborators [80] analyzed the chemical aspects and the antiepileptic activity of the substance 2-[(2,3-dimethylphenyl)amino]benzoic acid (Fig. **2**). The researchers initially performed analyses using an infrared spectrometer, Fourier Transform Raman Spectroscopy and computational calculations to investigate molecular geometry, vibrational attributions, Population density of molecular states, Frontier molecular orbitals, Hyperpolarizability calculations, among others. They also performed molecular docking using AutoDock 4.2 software and analyzed it with the Lamarckian genetic algorithm. Thyroxine binding globulin protein was obtained from the Protein Data Bank under code a 2XN3. The results showed that 2-[(2,3-dimethylphenyl)amino]benzoic acid was able to bind to the binding site and interact with the amino acids Gly366, Arg363, Val368, Ala318, Pro365, Leu310, and Leu306. In this way, the molecule was able to present antiepileptic activity.

**Fig. (3):**
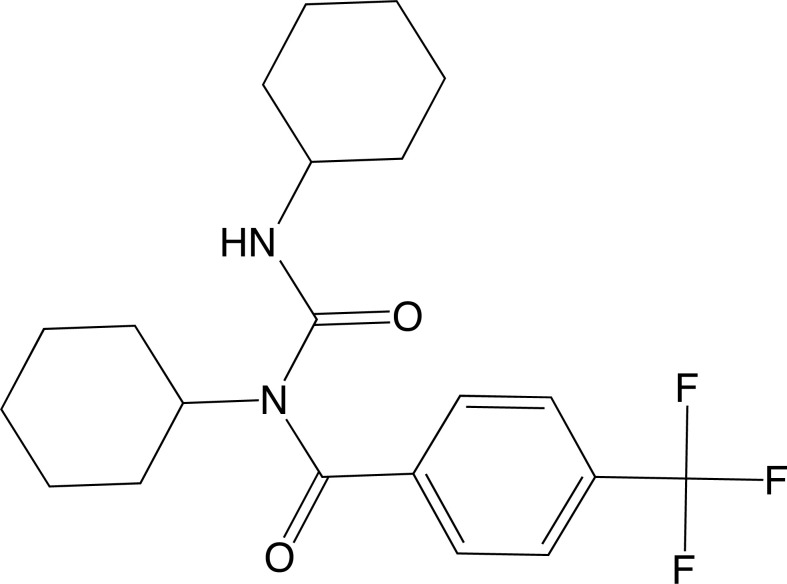
2D structure of the compound N-cyclohexyl-N-(cyclohexylcarbamoyl)-4-(trifluoromethyl)benzamide.

Another study on derivatives of benzoic acid investigated the antiepileptic activity of N-cyclohexyl-N-(cyclohexyl-carbamoyl)-4-(trifluoromethyl)benzamide (Fig. **3**) and its action on potassium channels. The researchers synthesized and analyzed the compound in terms of its structural characteristics. For pharmacological investigation, molecular modeling of the protein was performed using the protein sequence and crystal structures (PDB ID: 1JQ2, 1K4C, 1ORQ) of the voltage-dependent potassium channel, utilizing the BLAST and MODELLER programs for this process. Protein preparation for docking was conducted using the PrepWiz module of the Schrodinger suite, and molecular docking was performed using the Molegro Virtual Docker. Finally, ADMET parameters were analyzed on the admetSAR platform. The results showed that the compound exhibited a high binding energy value for the studied scores and interacted with the amino acid residues Arg307 and Arg459 through different types of bonds. In the ADMET tests, the compound demonstrated favorable parameters and low toxicity [81].

**Fig. (4):**
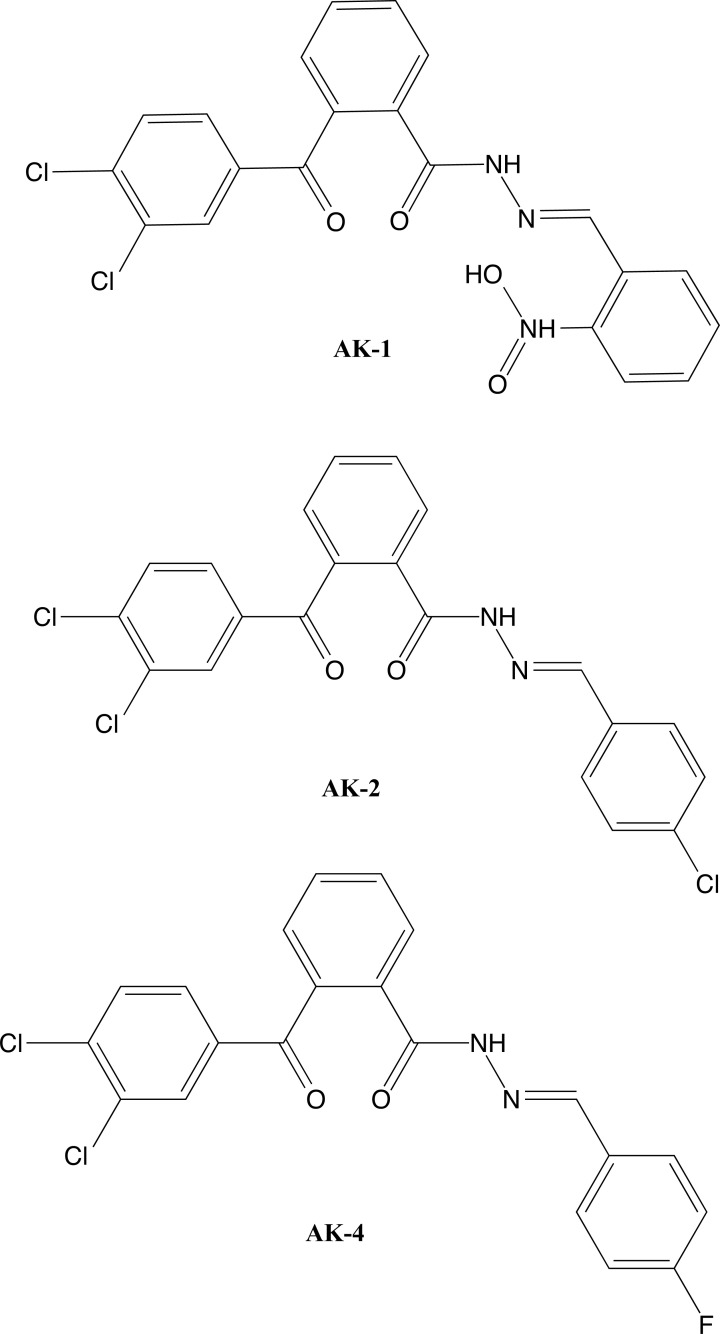
Structural formulas of compounds **AK-1**, **AK-2** and **Ak-4**.

Additionally, Kumar and Kumar (2020) [82] investigated the antiepileptic and antibacterial activity of new hydrazone derivatives. They designed and synthesized three compounds (Fig. **4**), which were analyzed through nuclear magnetic resonance spectroscopy and infrared spectrometry. Molecular docking was performed to screen compounds for the study of antiepileptic activity and anchoring was performed in the Auto-Dock Vina software. The crystalline proteins of c-Jun N-terminal kinase 3 (JNK3) were obtained from Protein Data Bank with codes 2ZDT and 2WAJ. Then, *in vivo* tests of maximum convulsion of electroshock and neurotoxicity and *in vitro* tests of antimicrobial screening were performed. The *in silico* results show that all compounds interacted with the target, and the antiepileptic activity of AK-2 and AK-4 was confirmed by *in vivo* studies. Finally, AK-1, AK-2, and AK-4 also showed antimicrobial character against *Escherichia coli* and *Staphylococcus aureus* bacteria.

### 4.2 Coumarins

**Fig. (5):**
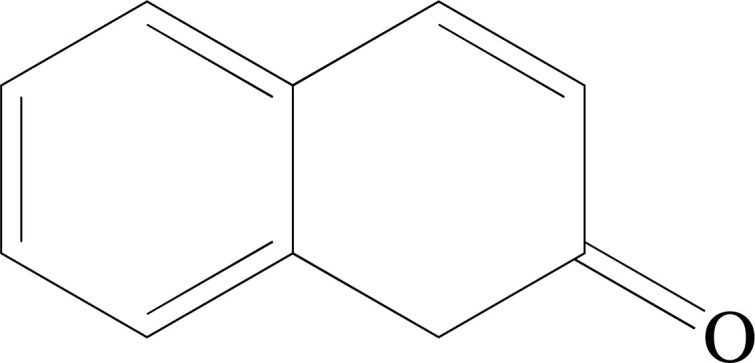
Chemical structure of coumarins.

Coumarins were first isolated by Vogel in 1820 from the species *Dipteryx odorata* (Cumaru) and are characterized as organic heterocycles studied as 1,2 benzopyrone (Fig. **5**) [83, 84]. Its chemical structure consists of a pyran ring fused to benzene with the pyrone carbonyl in position 2, so this class of compounds can also be called 2H-chromen-2-ones [84, 85].

**Fig. (6):**
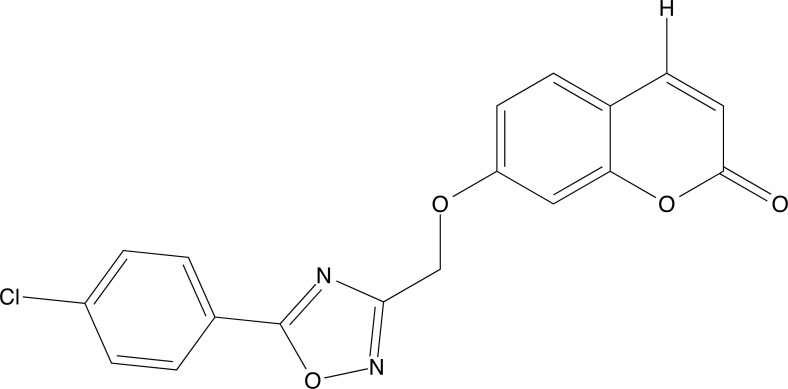
Structure of compound **3b**.

In search of new coumarin derivatives with anticonvulsant potential, new synthesis studies and preclinical analyses are constantly being carried out. Mohammadi-Khanaposhtani *et al.* (2019) [86] evaluated the anticonvulsant action of new coumarin-1,2,4-oxadiazole hybrids through *in silico* and *in vivo* studies. The derivatives were synthesized, and then *in vivo* analyses were carried out of the structure-activity relationship (SAR), neurotoxicity, and anticonvulsant potential by tests of convulsions induced by pentylenetetrazol (PTZ) and by maximum auricular shock (MES) in mice. *In silico* studies were also performed using molecular docking and drug likeness. Anchoring was performed using Autodock Tools (1.5.6), the selected protein was GABAA, and the drug-likeness prediction test was performed by ChemDraw Ultra 12.0, MarvineSketch 5.8.3 and Autodock Tools (ver.1.5.6) and permeability through the blood-brain barrier (BBB) was performed using the online BBB predictor. Some **3a**-**3m** substituted derivatives have been observed to have anticonvulsant and neuroprotective effects. Compound **3b** (Fig. **6**) showed the best results in molecular docking; it was able to connect to the GABAA binding site by interacting with the amino acid residues Phe77, Thr206, Tyr159, Tyr209, and Val211. In the drug-likeness study, 3b did not violate any of Lipinski's rules and is BBB permeable. Thus, compound **3b** has the potential to be an antiepileptic drug.

**Fig. (7):**
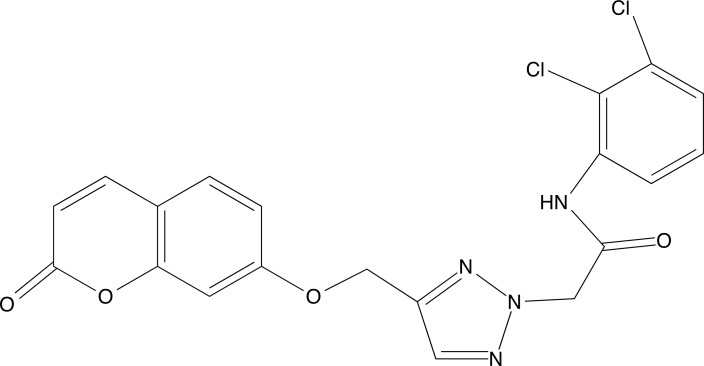
2D structure of the **8g** compound.

Characterization and molecular docking studies were performed with some coumarin-1,2,3-triazole-acetamide hybrid derivatives by Sepehri *et al.* (2020); a study was carried out that also evaluated the action of these derivatives on different pharmacological targets such as α-glycosidase (*α*-Gly), α-amylase (*α*-Amy), acetylcholinesterase (AChE), butyrylcholinesterase (BChE), human carbonic anhydrase I and II (hCA I and hCA II). The molecules were synthesized, and the authors found that one of these derivatives may have pharmacological potential for epilepsy. In the results with specific enzymatic inhibitors *in vitro*, compound **8g** (Fig. **7**) showed sub-nanomolar inhibition against the enzymatic targets hCA I and hCA II. Molecular docking was performed in the AutoDock software, and crystalline proteins of the enzymes hCA I (PDB ID: 4WR7) and hCA II (PDB ID: 5AML) were also obtained and later prepared in AutoDock Tools 1.5.6 and the molecule was drawn using MarvinSketch 5.10.4. And finally, the anchors were visualized in Discovery Studio 2019 Client. The results showed that compound **8g** strongly interacted with the binding site of hCA I and hCA II, having in common the interaction with the amino acid residue Gln92, the other interactions with hCA I were with Ala121, Ala135, His200, Leu131, Leu198, Pro202, Thr199 and Zn301 and with hCA II were with Ala65, His94, Leu141, Leu198, Trp209, Val121, Val143, and Zn265 [87].

**Fig. (8):**
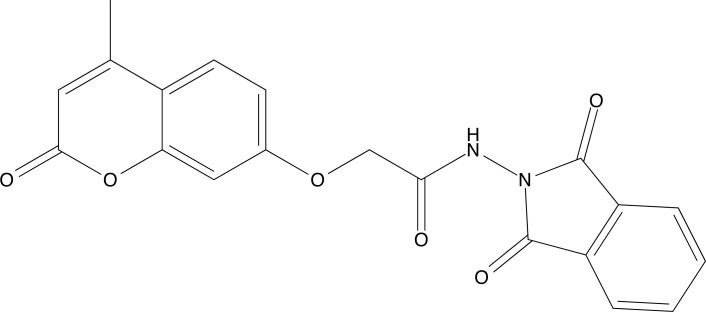
2D structure of compound **6**.

Abd-Allah *et al.* (2020) [88] also evaluated the anticonvulsant potential of a series of synthesized coumarin derivatives. In their study, benzopyrone molecules were replaced and divided into different classes, from **6** to **16b**, and the anticonvulsant potential was evaluated through *in vivo* tests of chemical induction of epileptic seizures. The researchers also performed *in silico* pharmacokinetic and molecular docking studies for the target γ-aminobutyric acid aminotransferase (GABA-AT). The crystalline protein of GABA-AT was obtained from RCSB PBD under code 1OWH. CDOCKER protocol was used for anchoring and minimization of molecules. The Discovery Studio 2016 was used for molecular modeling. Moreover, pharmacokinetic parameters were evaluated using the SwissADME platform. In this way, compound **6** (Fig. **8**) protected the animals against epileptic seizures and proved not to be neurotoxic, in molecular docking it had the best chemical interaction, interacting with the amino acid residues Arg192, Cys135, Glu270, Gly136, His206, Ile72, Lys329, and Phe189, in addition to other residues through van der Waals-type interactions. Finally, compound **6** showed good bioavailability, did not violate any Lipinski rule, and exhibited the ability to be easily absorbed, therefore a potential anticonvulsant drug.

**Fig. (9):**
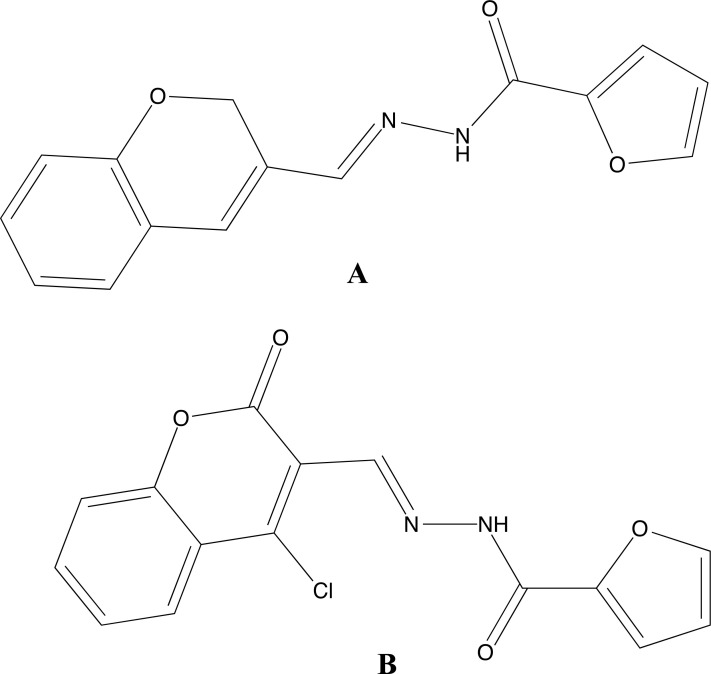
2D structure of (**A**) compound **8b**; (**B**) compound **4b**.

In another study carried out by Angelova *et al.* (2017), a series of coumarin derivatives of the type chromene-based aroyl hydrazones were synthesized and evaluated for their anticonvulsant and neuroprotective potential through *in silico*, *in vitro*, and *in vivo* methodologies. In the study, for the tests of seizures induced by maximum auricular electroshock and the test of seizures induced by PTZ, the 2H-chromene **8b** derivative (Fig. **9A**) was the one that presented the best protection to the animals, while the methoxyphenyl-substituted derivative **4b** (Fig. **9B**) was the most active in the 6Hz test. Computational studies were performed using Molecular Operating Environment (MOE, version 2016.08) software for molecular docking and Marvin 16.2.8.0 for pharmacokinetic analyses. In addition, the GABAA crystalline protein was taken from the Protein Data Bank under code 4COF. The results of *in silico* tests showed that compounds **8b** and **4b** interacted with the same amino acid residues Leu99, Glu155, Gly158, Phe200, Thr202, Tyr97, Tyr157, and Tyr205. Furthermore, **4b** interacted with Ala201. The pharmacokinetic parameters demonstrated that these compounds have good bioavailability and permeability to BBB, being excellent drug candidates.

**Fig. (10):**
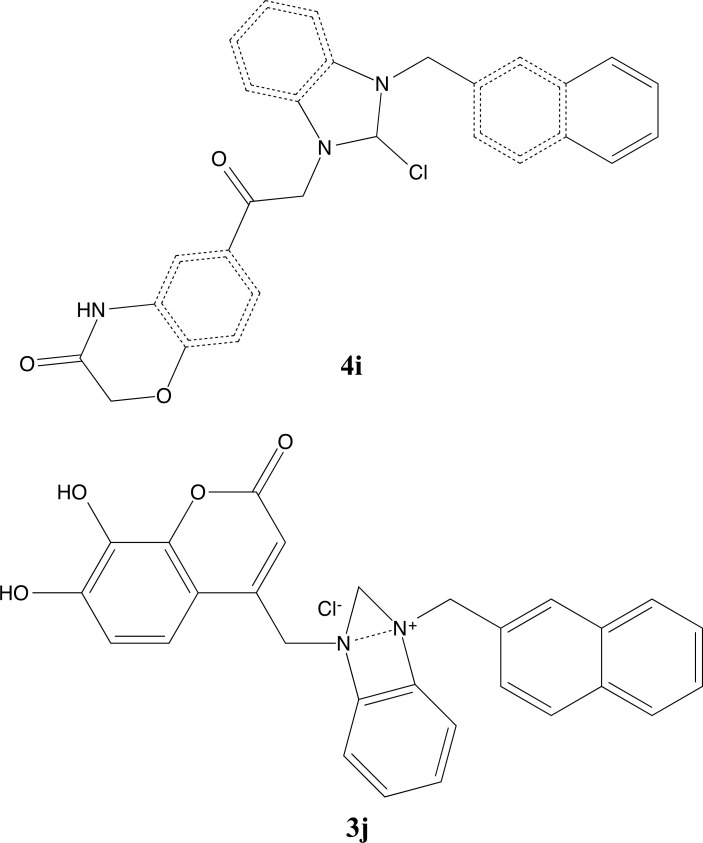
2D structures of compounds **4i** and **3j**.

As the inhibition of the enzyme human carbonic anhydrase (hCA) is one of the mechanisms that may be involved in the anticonvulsant action of a drug, Karatas *et al.* (2016) [89] conducted a study with twenty-four new synthesized coumarin derivatives. They evaluated the chemical interactions between these derivatives and hCA through molecular docking, its *in vitro* inhibition, and the *in vivo* anticonvulsant potential through MES and the ScMet-induced seizures test. Of the analyzed compounds, all inhibited both hCA I and hCA II, and derivative **4i** (Fig. **10**) was the one that showed the best anticonvulsant and neuroprotective potential *in vivo*. For the molecular docking, the hCA II crystal proteins were obtained from the RCSB PDB under the 3EFT code and modified in the ADT package version 1.5.6rc3 program. The compounds were minimized and optimized in the GAMESS software. Protein-ligand docking was performed in AutoDock 4 and interactions were visualized in Discovery Studio 4.0 Client. Although compound **4i** (Fig. **10**) showed the best results in animal tests, it was not selected for the *in silico* test, so compound **3j** (Fig. **10**) showed the best binding energy and interacted with the amino acids Gln92, Leu198, Phe 131, Pro202, Thr199, Val121, and Zn262. The authors, therefore, concluded that the synthesized compounds acted similarly to other studies involving coumarins, protecting against epileptic seizures.

**Fig. (11):**
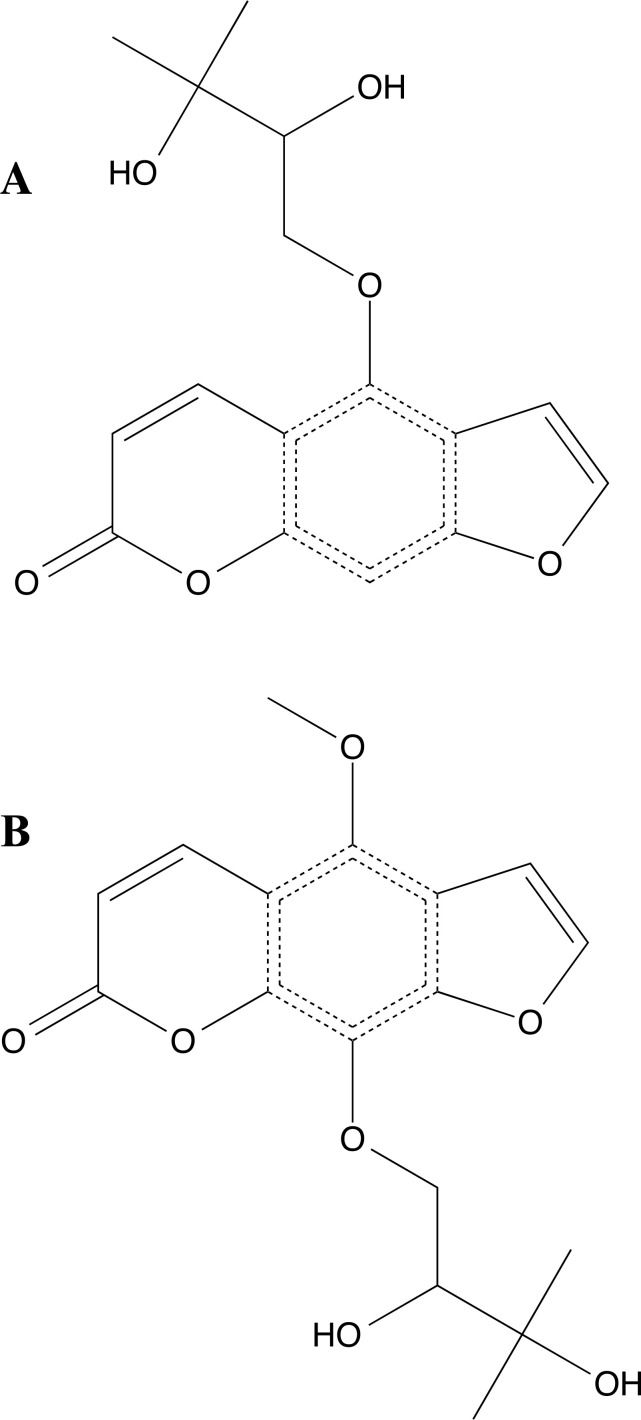
2D structures of oxypeucedanin hydrate (**A**) and byacangelicin (**B**).

Kozioł *et al.* (2021) [90] also carried out a comparative *in vivo* study of the anticonvulsant effect of a series of new coumarin derivatives, but this time in Zebrafish (*Danio rerio*) by testing the induction of epileptic seizures by PTZ in Zebrafish larvae. Eighteen derivatives were evaluated, of which seven were reduced in at least 29% of the crises and, in four of them, over 50%. The researchers then selected the derivatives oxypeucedanin hydrate (Fig. **11A**) and byacangelicin (Fig. **11B**) to evaluate their *in silico* interaction with GABA transaminase (PDB ID:1OHV). Molecular docking was performed using Molegro Virtual Docker v.6.0, and molecules were built using HyperChem 6.05 software. The result demonstrated that both molecules present good molecular interaction with the target and interacted with the same amino acid residues Arg422, Asn423, Glu270, and Lys203.

Seeking to investigate the action of coumarin derivatives on adenosine receptors (AR), aiming to evaluate the biological potential against diseases such as Alzheimer's, Parkinson's, Epilepsy, and Schizophrenia, Vazquez-Rodriguez *et al.* (2020) [91], synthesized substituted hybrids (1-8) and evaluated the interaction with adenylyl cyclase (hA2B) and radioligands (hA1, hA2A and hA3), to investigate the interaction with human AR (hAR). The hydroxy-substituted hybrids showed a greater affinity for hA1, and the methoxy-substituted ones had greater affinity for hA3. In the *in silico* studies, pharmacokinetic parameters were performed using different theoretical calculations, and molecular docking was performed in the Glide SP mode of the Schrodinger package. In the studies of Absorption, Distribution, Metabolism, and Excretion (ADME), none of the eight molecules analyzed showed any violation of Lipinski's rules, in addition to showing good absorption. Finally, in the molecular docking, it was possible to observe a good chemical interaction between the hydroxy- and methoxy-substituted derivatives and the hAR.

**Fig. (12):**
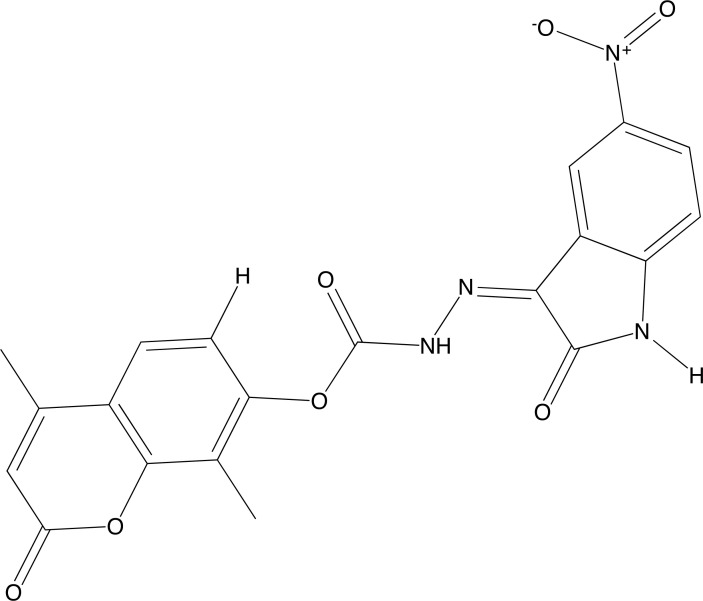
Compound M5N.

Adsule, Chabuskwar, and Nanaware (2021) [92] researched the anti-inflammatory and anticonvulsant potentials of various substituted coumarin derivatives. They evaluated the anticonvulsant potential *in vivo* using the maximum auricular electroshock (MES) test and, using molecular docking, tested the chemical interaction of the derivatives with hCA for anticonvulsant potential and the enzyme cyclooxygenase (COX) for anti-inflammatory potential. The computational study was performed using the V Life software 3D ultra 8.0. The compound M5N (Fig. **12**) showed the best results in the fit with the hCA II protein (PDB ID:3F8E), interacting with the amino acids Asn62, Asn67, and Ile91 and in the fit with the COX I protein (PDB ID:3PGH), interacting with Arg120 and Tyr355. It was also observed in the study that most derivatives showed protective potential in MES at a dose of 200 mg/kg, and more than twenty of these molecules showed protection of at least 67%. In the SAR study, it was observed that the replacement of the isatin nucleus is fundamental for the improvement of the potential.

### 4.3 Simple Phenolic Compounds

**Fig. (13):**
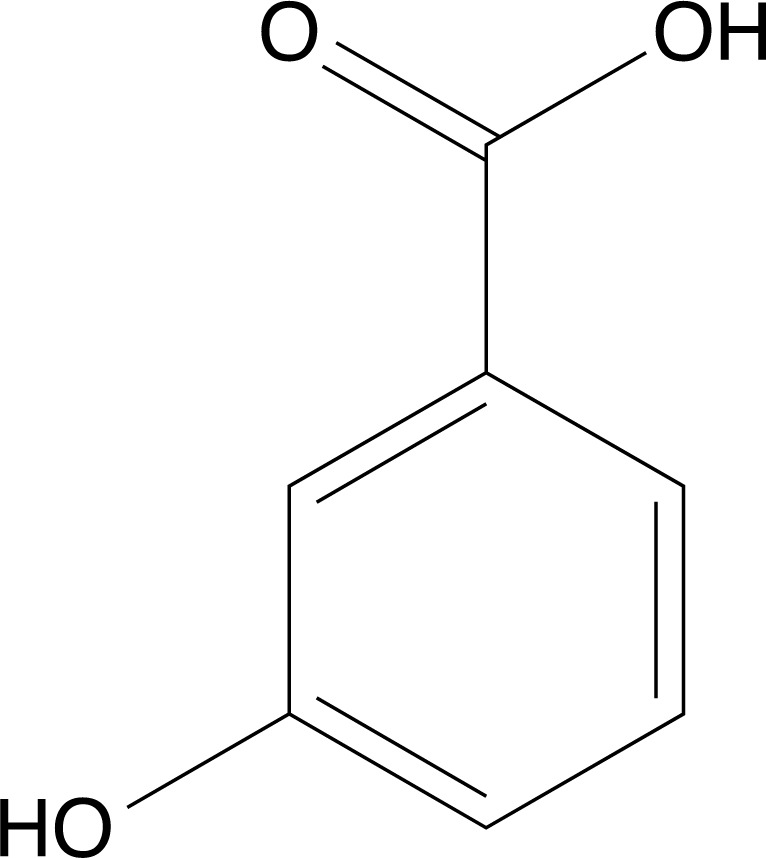
Chemical structure of Simple phenolic compounds.

Phenolic compounds are secondary metabolites abundantly synthesized in the plant kingdom and are widely studied [93]. They act mainly as defense agents in response to stress caused to fruits and vegetables, giving them astringency, color, flavor, and aroma [94]. In its structure is found at least one aromatic ring with one or more hydroxyl groups attached [95]. They can be classified as simple phenols or polyphenols, and the number of phenol subunits determines the classification of compounds: simple phenols, such as those derived from benzoic acid and cinnamic acid, contain only one phenol subunit, while polyphenols, such as flavonoids have two or more subunits (Fig. **13**) [96].

**Fig. (14):**
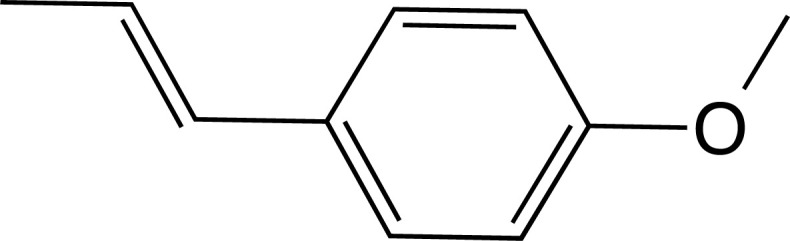
2D chemical structure of the trans-anethole (TAN) compound.

Guedes *et al.* [97] published an article addressing the action of *trans*-anethole (TAN), promoting the anticonvulsant effect in mice. Experiments on the induction of epileptic seizures by MES and pentylenetetrazole (PTZ) were carried out. In this study, the anticonvulsant activity of TAN (Fig. **14**) was evaluated using the PTZ-induced seizure test in animals. The results showed that TAN exhibited a protective effect similar to diazepam, a standard drug. Additionally, TAN did not result in deaths in the treated groups, unlike another similar compound, isopentyl ferulate. These results indicate the promising potential of TAN as an anticonvulsant agent, possibly acting on GABAergic transmission.

**Fig. (15):**
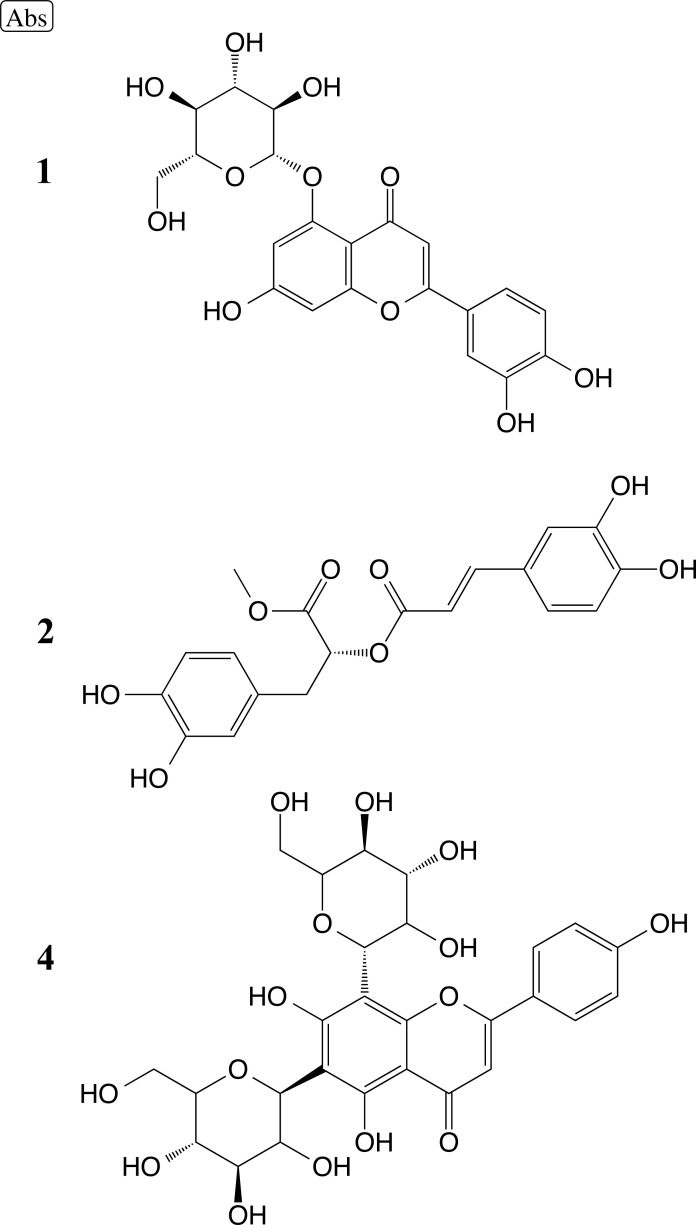
2D structures of luteolin 5-O-β-glucoside (**1**), methyl rosmarinate (**2**), vicenin 2 (**4**).

Rauf *et al.* [98] conducted a study involving eight plant phenolic compounds to assess their inhibitory activity against carbonic anhydrase-II (CA-II) and urease using microtiter assays. They also performed molecular docking simulations using the i-GEMDOCK and Autodock Vina programs, with the protein PDB ID 1V9E as the target. The results showed that compounds 1 (luteolin 5-O-β-glucoside), 2 (methyl rosmarinate), and 4 (vicenin 2) (Fig. **15**) exhibited notable inhibitory action above 50% against CA-II and also displayed moderate inhibitory capacity against urease. While the tested compounds have shown low toxicity in normal cells in previous studies. In the results of molecular docking, the authors identified several important residues involved in ligand-receptor interactions: His2, Val59, Asn61, His63, Asn66, Glu68, Gln91, His93, Val120, Lys168, Gly169, Leu196, Thr198, and Pro199. Compounds **1**, **2**, and **4** show potential to be evaluated as potential CA-II inhibitors in future research.

### 4.4 Flavonoids

**Fig. (16):**
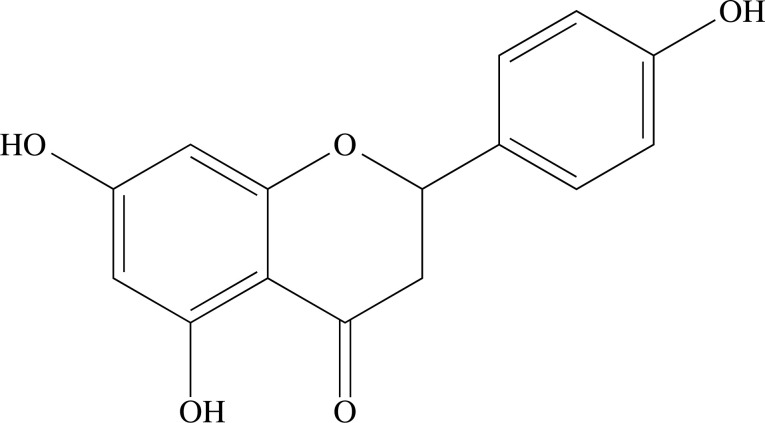
Chemical structure of flavonoids.

Flavonoid is the name given to compounds that have a skeleton of 15 carbons arranged in three rings (C6-C3-C6), which consist of two phenyl rings (A and B) connected by a three-carbon bridge (ring C) [99, 100] (Fig. **16**). Flavonoids are a class of secondary metabolites that have a great diversity of compounds and great importance in several areas, as well as having a very wide occurrence in the plant kingdom, being seen in all plant phyla [101-104]. They are classified into eight groups of main compounds according to the different structural arrangements, which correspond to: flavones [105], flavonols [106], flavanones [107], flavanonols [108], flavanols [109], anthocyanins [110], chalcones [111], aurones [112] e neoflavonoids [104, 113-115].

**Fig. (17):**
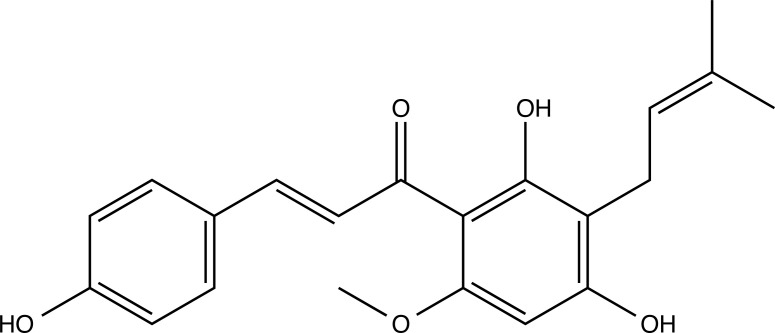
2D chemical structure of the compound xanthohumol.

Aydin *et al.* [116] conducted a study involving the main prenylated chalcone from hop cones, xanthohumol (Fig. **17**), which was subjected to molecular docking against the isoenzymes hCA I and II, as well as AChE and BChE. Catechin, quercetin, p-coumaric acid, and phenol were used as positive controls in the study, and the software Maestro was utilized for the docking analysis. Xanthohumol exhibited the highest inhibitory effects among all tested compounds, particularly against CA isoenzymes, showing an effective inhibition constant (KI) value of 0.049 µM for hCA II. The flexible chemical structure of xanthohumol, without a ring between the phenolic rings, may account for its strong inhibitory activity compared to other evaluated compounds. Xanthohumol displayed important interactions with amino acids Gln92, His67, His94, Phe91, and Thr199 in the hCA II binding site.

Wang *et al.* [117], in this study, the authors conducted research using network pharmacology and molecular docking to explore the mechanism of the Kangxian decoction (KXD) for treating epilepsy. KXD is a combination of six traditional Chinese medicines, which include *Acorus tatarinowii* (Shichangpu), *Pinellia ternate* (Banxia), *Panax notoginseng* (Sanqi), *Gastrodia elata* (Tianma), *Uncaria* (Gouteng), and *Pheretima* (Dilong). This composition is known for its beneficial properties in eliminating phlegm and promoting blood circulation. In this study, the authors utilized molecules from the Encyclopedia of Traditional Chinese Medicine (https://www.tcmip.cn/ETCM) and imported them into the SwissADME platform to predict some important pharmacological parameters and propose new bioactive. Molecular docking simulations were performed using the AutoDock Tools 1.5.6 program against the proteins with PDB IDs 3THJ, 5IKT, 5TH6, 1IKL, and 1MFL. Utilizing network pharmacology and molecular docking, the authors concluded that they identified the main components of KXD and the therapeutic targets associated with epilepsy.

**Fig. (18):**
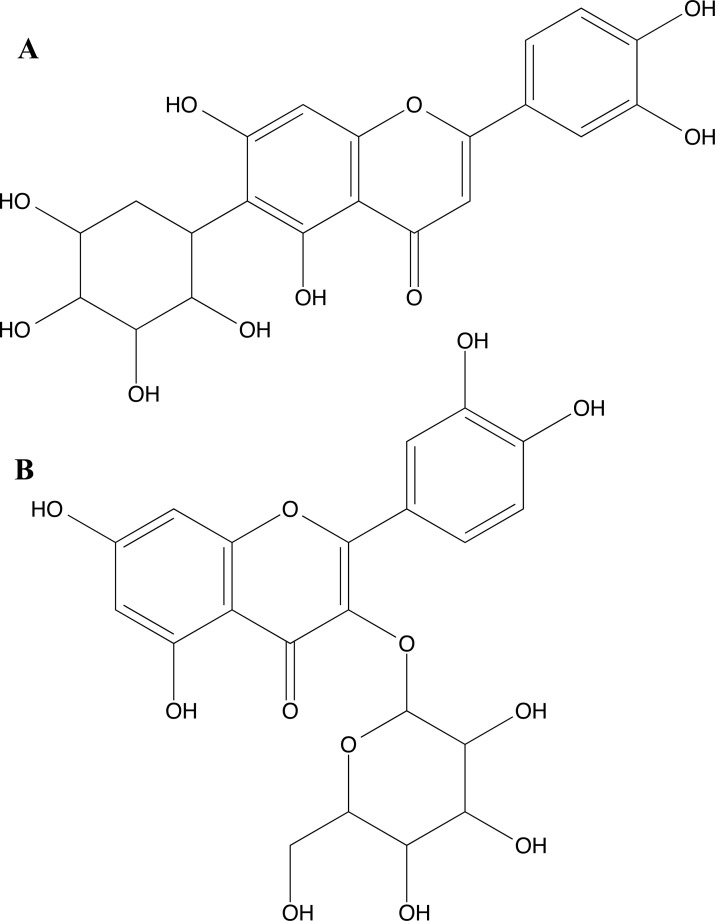
2D structures of homoorientin (**A**) and quercetin-3-O-galactoside (**B**).

Silva *et al.* [118], in this study, investigated the anxiolytic and anticonvulsant effects of the ethanolic extract of Combretum lanceolatum Pohl. Leaves in adult zebrafish. Tests were carried out to determine the concentration of total phenols, identify flavonoids by HPLC and evaluate the *in vitro* antioxidant activity. Additionally, the toxicity test and the mechanism of action at the GABAA receptor and hCA II were examined. The flavonoids identified in the extract of *C. lanceolatum* were orientin, quercetin-3-o-galactoside, vitexin, isovitexin, rutin, and homoorientin. The authors also performed molecular docking using Autodocktools and AutoDockVina software. The docking of homoorientin (Fig. **18A**) and quercetin-3-O-galactoside (Fig. **18B**) compounds with GABAA (PDB ID:6HUP) showed some important interactions, such as Tyr753, Phe772, Thr837, Asn755, Asp887, Ser890, His1137, Lys1191 and Tyr1195. And the compounds homoorientin and isovitexin with hCA II (PDB ID 3F8E) showed interactions with the amino acid residues Glu69, Ile91, Gln92, Asn62, Arg58, His64, Asp72, Thr200, and Pro201. The authors concluded that this plant has anxiolytic and antiepileptic properties.

**Fig. (19):**
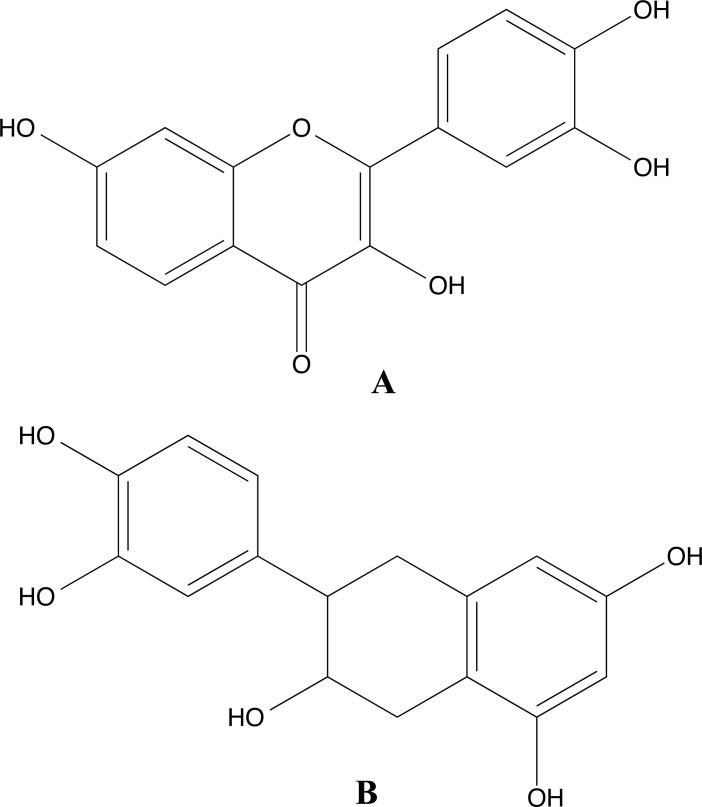
2D chemical structure of the compounds fisetin (**A**) and 6-(3,4-dihydroxyphenyl)-5,6,7,8-tetrahydronaphthalene-1,3,7-triol (**B**).

Aditama *et al.* [119] executed a screening of natural product compounds using a molecular docking method to identify potential inhibitors of phenolics and flavonoids. Among the compounds tested, two compounds stood out: fisetin (Fig. **19A**) and 6-(3,4-dihydroxyphenyl)-5,6,7,8-tetrahydronaphthalene-1,3,7-triol (Fig. **19B**), as they showed promise as potential inhibitors of the enzyme CAII. The inhibitors were analyzed by preparing and minimizing their molecular structures using the program Marvin Sketch with the MMFF94 force field. To evaluate the interaction of the inhibitors with the enzyme CAII, molecular docking was performed using the GOLD software, employing a genetic algorithm to find the best binding position within the active site of CAII against PDB ID 2Q38. Additionally, the authors conducted molecular dynamics simulations using AMBER under the AMBER 2012 force field for 2 ns at 1 atm and 310 K. The study of the computational simulations pointed to a promising inhibitory effect of some molecules among those studied, highlighting specific interactions with the amino acid residues Asn62, Asn67, His199, His94, His96, Gln92, and Thr199.

**Fig. (20):**
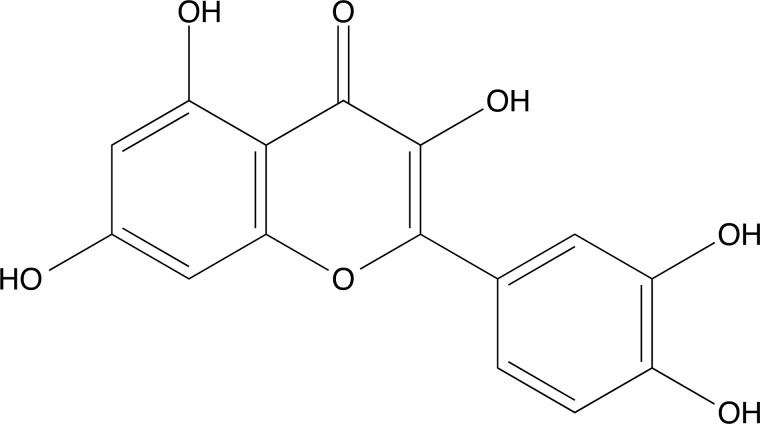
2D structures of quercetin.

Redford *et al.* [120] published a study indicating that quercetin (Fig. **20**) is an atypical activator of the KCNQ potassium channel. For the study, they conducted an extraction of pickled capers (*Capparis spinosa*). This extract was tested on *Xenopus laevis* oocytes, both uninjected and expressing KCNQ1 or KCNQ2/3 potassium channels. The electrophysiology technique known as two-electrode voltage clamp (TEVC) was used to perform the analyses. For the binding site prediction, molecular docking simulations using SwissDock with CHARMM 58 force fields against KCNQ1, KCNQ1/KCNE1, and KCNQ1/KCNE3. The TEVC results showed that quercetin acted by activating KCNQ, and in molecular docking, it was observed that the molecule connects to residues Arg231 and Arg228.

Ahmed *et al.* [121], in a published article, evaluated the therapeutic potential of kaempferol, quercetin, and catechin for the treatment of chronic epilepsy in a rat model. Behavioral tests were performed, including the light and dark test, elevated plus maze, rotarod test, infrared actimeter, beam walk test, sucrose preference test, and forced swim test, along with histopathological evaluations. Molecular dynamics simulations were performed for these molecules against the SV2A receptor (PDB ID 4V11), sodium channel (PDB ID 6AGF), and GABAA receptor (PDB ID 6D6T) using PyRx, PyMol, and Autodock software. Molecular docking results showed that phytoflavonoids interacted with the SV2A receptor through the amino acid residues Lys333, Ala396, and Leu281. The authors concluded that the potential binding of phytoflavonoids to the SV2A receptor suggests its inhibitory capacity. Furthermore, biological studies have revealed the ability of these compounds to modulate the immune response, indicating that they may play a crucial role as a therapeutic alternative in the treatment of epilepsy.

**Fig. (21):**
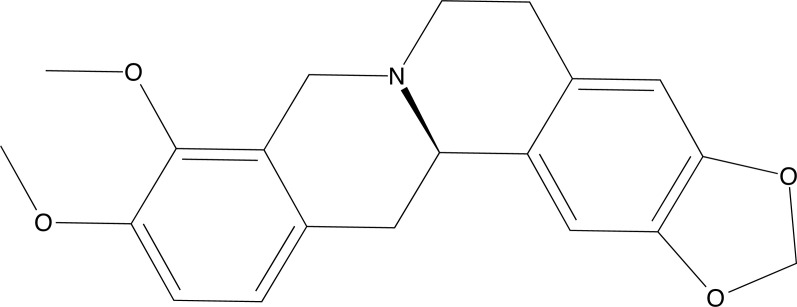
2D structures of (R)-canadine.

Huang *et al.* [122], in their work, studied the involving network pharmacology to explore the multi-target antiepileptic mechanism of Rhizoma Coptidis. In this research, a prediction was initially made using genes to analyze the main targets of epilepsy, then the main bioactive components of the plant were collected from the Traditional Chinese Medicine Systems Pharmacology Database, then a network was created between the words Epilepsy and Rhizoma coptidis. The results showed that the targets (PDB ID 2UZS), VEGFA (PDB ID 6D3O), IL6 (PDB ID 1ALU), and TP53 (PDB ID 3DCY) were connected with both data. Therefore molecular docking was performed with these proteins and the bioactive components of Rhizoma Coptidis. For this, the AutoDock program was used. The molecular docking simulations revealed that quercetin and (R)-canadine (Fig. **21**) exhibited favorable interactions with the main targets. The results suggest that the investigated targets have great relevance in triggering epileptic seizures and, therefore, are possible pharmacological targets.

## 5 EXPERIMENTAL

### 5.1 Obtaining the Molecules

A total of 468 phenylpropanoids were selected from the literature. For the execution of the models, molecules with pharmacological activity were obtained from the ChEMBL chemical structure database (https://www.ebi.ac.uk/chembl/), for the proteins: gamma-aminobutyric acid A (GABAA), N-methyl D-aspartate (NMDA), voltage-gated sodium channel (NaV), gamma-aminobutyric acid 1 (GAT-1), voltage-gated potassium channel of the Q family (KCNQ), voltage-gated calcium channel T-type (CaV), GluR1, GluR2, GluR3, and GluR4, the last four are subunits of the protein alpha-amino-3-hydroxy-5-methyl-4-isoxazole-propionic acid (AMPA), under the codes and activities represented in Table **SM1** (Supplementary Material).

### 5.2 Database Preparation

The data selected from CHEMBL were prepared in the Microsoft Office Excel program with columns, namely molecular weight, SMILES, and concentration value or activity constant of the molecules, for later calculation and analysis in the descriptors. For concentrations with units expressed in nanomolar (nM), they were transformed into molar (M), then into pIC50, pCKI, or pEC50, calculated using a negative logarithm unit (-log) [123-125]; duplicates were also removed. Molecules with the highest values for activity were classified as “A” and “I” for inactive molecules. In addition, outliers were removed, creating a margin of distance between the values of active and inactive molecules.

Finally, the bioactive molecules were standardized using the ChemAxon Standardizer 20.19.0 program. As input data, the SMILES of the molecules after the addition of explicit hydrogens, 3D clean, aromatization, and removal of salts were used.

### 5.3 Calculations of Molecular Descriptors

Molecular descriptors were generated to obtain information regarding the analyzed molecules, which are generated descriptors of biological activities, structural, physicochemical, surface topology, conformational states, and others. These descriptors were calculated by different logarithms through the programs Dragon (D) [126], CDK v1.4.8 (C) [127], and RDKIT (R) [128]. Molecules in SDF format generated in Standardizer 20.19.0 were imported, the data obtained were treated for analysis of empty ballots in Microsoft Office Excel, and later, the spreadsheets obtained were used for the development of Ligand-based virtual screening (LBVS) models.

### 5.4 Building Ligand-based Virtual Screening Models

The prediction model aims to predict biological activities, so the spreadsheets formed from the bioactive molecules of the targets and phenylpropanoids in the molecular descriptors were imported into a workflow developed for drug delivery in the Konstanz Information Miner (KNIME) Analytics Platform 4.4.0 software [129].

Algorithms were used to build the model utilizing a classification method, such as Weka 7.0 and Random Forest, which is based on the formation of random decision forests through the generation of several decision trees with training using the method out-of-bag. This method consists of combining learning models to classify the accuracy by the KNIME program [130]. In addition, to confirm the reliability of the model, statistical data were collected for precision (PR), sensitivity (SE), specificity (SP), accuracy (AC), and the results of the Matthews Correlation Coefficient (MCC) (Table **2**); using the mathematical expressions (Table **SM2**, Supplementary Material): true positive (VP), false positive (FP), false negative (FN), true negative (VN), such as the ROC curve (Receiver Operating Characteristic) and prediction and percentage of activity. Models that present statistical values ≤ 0.6 were discarded, as they do not have predictive quality. Finally, a consensus calculation of activities (ATV C) was carried out with all the results obtained from the phenylpropanoids the descriptors generated in the three software used to reduce the error, molecules that present values ≥ 50.0% will be considered active (A) and the others inactive (I) [131-133].

### 5.5 Oral Absorption, Toxicity Risks and Blood-brain Barrier Permeation

Oral absorption (%ABS) and toxicity analyzes were performed using the OSIRIS Data Warrior 5.0 software [134]. And analyzes were executed on the permeability of the blood-brain barrier on the SwissADME platform (http://www.swissadme.ch/index.php). Oral absorption was analyzed by two predictive parameters. Initially, the percentage of the absorption rate of the phenylpropanoids that passed the LBVS model was calculated based on the total topological surface area (TPSA) using the equation:

Subsequently, Lipinski's rule of 5 was applied, which consists of determining the greatest chances of oral bioavailability using five parameters: partition coefficient (clog P) ≤ 5, molecular weight ≤ 500 daltons, number of hydrogen bonding acceptors ≤ 10 and number of hydrogen bond donors ≤ 5 [135, 136]. Active phenylpropanoid molecules that showed an absorption rate < 80% and violated any of Lipinski's rules were discarded.

Toxicity risks were predicted according to mutagenicity, carcinogenicity, tissue irritability, and toxic effects on the reproductive system. Molecules that do not present any of these types of toxicity were chosen to present greater safety. In addition, the toxicity of the metabolites of the selected molecules was analyzed [137].

### 5.6 Liver Metabolism Prediction

For the analysis of the metabolites, a model was built that predicts the probable hepatic metabolites of the phenylpropanoid molecules approved in the previous models, for which the METASITE 6.0 software was used [138]. The enzyme used to carry out the tests is cytochrome p450 2D6, found in the liver. The metabolites were generated and analyzed regarding the parameters: the probability of these metabolites being formed is greater than 50%, and molecular mass is greater than 100 g/mol. All approved metabolites were checked for cytotoxicity, and only those that did not show any toxicity were selected for molecular anchoring [139,140].

### 5.7 Molecular Docking

Initially, the structures of the phenylpropanoids approved in the other tests and of the control drugs (Fig. **SM1**, Supplementary Material) were drawn in Marvin Sketch 21.13 and optimized in the HyperChem 8.0.6 software (RMS 0.1 kcal/mol/Å) in standard configuration, using the field method of strength of molecular mechanics MM+ and the semi-empirical quantum method AM1 (Austin Model 1), applied to small molecules and antioxidant substances [141-143].

The crystalline proteins of the targets with active molecules were obtained from the RCSB Protein Data Bank (PDB) (http://www.rcsb.org/pdb/home/home.do), the targets being: AMPA (PDB ID: 1FTL) resolution of 1.80 Å and X-ray diffraction method [144]; CaV (PDB ID: 6KZP) resolution of 3.10 Å and electron microscopy method [145]; GABAA (PDB ID: 6D6T) 3.86 Å resolution and X-ray diffraction method [146]; GAT-1 (PDB ID: 7SK2) resolution of 3.82 Å and electron microscopy method [147]; and NMDA (PDB ID: 5VIH) resolution of 2.40 Å and X-ray diffraction method [148]. Anchoring of phenylpropanoids and control drugs with proteins was performed using the Molegro Virtual Docker (MVD) software. The following parameters were used to configure the function of the binding energy scores: MolDock Score and PLANTS Score, of which a consensus was reached through the arithmetic mean with the binding energy values obtained to decrease the error [149-151]. A cut-off point was established for binding energy < -8.0 kcal/mol; GRID presented a resolution of 0.3 Å and a spherical radius of 15 Å. In the MolDock Score, the evaluation of the binder was determined in terms of internal ES, internal Hbond and Sp2-Sp2 torsion and in the PLANTS Score, hydrogen was evaluated in terms of torsion; the algorithm used to carry out both parameters was MolDock Simplex Evolution (MolDock SE) in which a number of runs of 10 and maximum iterations of 1500 were performed [152].

To validate the technique, redocking was performed, values for Root Mean Square Deviation (RMSD) were analyzed, and an RMSD cut-off value ≤ 2.0 Å was stipulated [153]. Another parameter evaluated was the ligand-protein interactions, for which 2D and 3D images were visualized using the Biovia Discovery Studio Visualizer 19.1.0.18287 program (https://discover.3ds.com/discovery-studio-visualizer-download).

## 6 RESULTS

### 6.1 Ligand-based Virtual Screening Models (LBVS)

The ligand-based virtual screening models developed in KNIME obtained their reliability and significance analyzed through statistical calculations for the parameters precision, sensitivity, specificity, and accuracy, in addition to the Matthews Correlation Coefficient (MCC) and ROC curves values to evaluate performance and robustness of the developed model. It was then observed that there were statistically significant results for all statistical analyses, characterizing a reproducible and valid model. However, the model for the KCNQ target could not be executed due to the presence of errors, partly due to the small sample of data found for the development of the LBVS.

The values for the parameters (Table **SM3**, Supplementary Material) in the test model varied as follows: from 0.83 to 0.89 for precision, from 0.73 to 0.91 for sensitivity, from 0.79 to 0.90 for specificity, and from 0.80 to 0.88 for accuracy. For the coefficient of MCC, results were obtained from 3 models with values ≥ 0.70 and another 3 models ≥ 0.60.

Furthermore, the prediction models were evaluated using ROC curve graphs (Figs. **SM2** and **SM3**, Supplementary Material). Thus, the ROC curves for both test sets and cross-validation showed values > 0.93 for the AMPA model, > 0.92 for the CaV model, > 0.84 for the GABAA model, > 0.90 for the GAT-1 model, > 0.89 for the NaV model, and > 0.88 for the NMDA model. In this way, the developed prediction models had values > 0.8, and the probability of these models selecting molecules that present biological activity for the determined targets was high.

Once the prediction models showed adequate performance and reliability. The 468 phenylpropanoids molecules were analyzed in the LBVS models using the three software programs to generate the molecular descriptors, which evaluate different chemical and biological parameters for drugs with activity in epilepsy. In this model, the consensus was reached on the results of the possibility of activity for the 3 software (Dragon, CDK, and RDKIT), and the active molecules that resemble the properties present for each target prediction model were selected. In this way, several molecules exhibited possible biological activity, presenting a reliable domain (Table **1**), proving the feasibility of the test, since it validated the study as reproducible.

The results presented that 8 molecules had activity against AMPA, 5 molecules activity in favor of CaV, 20 molecules in favor of GABAA, 3 molecules against GAT-1, and 20 molecules against NMDA. The NaV did not show any active molecule, and the probability range was between 2.0% and 25.04%. After analyzing the model, all molecules that exhibited activity were separated, and duplicates were removed, totaling 53 molecules. This allowed us to state the significance of these 53 phenylpropanoid molecules that showed more than 50% activity in the selected targets.

### 6.2 Oral Absorption and Toxicity Risks

In the ADMET, 21 molecules were identified as active based on these predictions. It was observed that the compounds (Table **SM4**, Supplementary Material) exhibited partition coefficients (octanol/water) ranging from 3.37 to 4.90, indicating moderate lipophilicity. Upon analyzing the water solubility coefficient of these same compounds, it was observed that the values ranged from -5.48 to -3.96, signifying moderate solubility. However, the potential negative effects of this prediction can be minimized if confirmed through benchtop solubility testing with the aid of appropriate pharmaceutical formulation.

Based on the total polar surface area, oral absorption rates were calculated for all the compounds analyzed. For all of them, the absorption rate was above 86%, suggesting that these compounds have the potential to be administered orally.

Besides that, the compounds did not show any toxicity risks in any of the cytotoxic parameters considered in this research. The results about the bioavailability potential of these compounds showed that none of them violated any of Lipinski's 5 rules. Furthermore, it was observed that all 21 molecules have the possible ability to cross the BBB.

### 6.3 Liver Metabolism Prediction

The 21 molecules were imported into the METASITE 6.0 program for the prediction of Hepatic metabolites. Of these compounds, only 12 (Table **SM5**, Supplementary Material) reached the criteria and were selected for molecular docking. The percentage of possible formation of these metabolites ranged from 53.65% to 100.00%, and the molecular weight of these metabolites ranged from 225.290 g/mol to 365.384 g/mol.

These metabolites were subjected to cytotoxicity analysis, assessing the four parameters discussed earlier: mutagenicity, carcinogenicity, reproductive toxicity, and irritability. The metabolites that did not present any risk of toxicity were considered.

Furthermore, the metabolic reactions responsible for the formation of metabolites were analyzed. The metabolites of the phenylpropanoids (Table SM6, Supplementary Material) TR157, TR117, and TR122 have a 30% chance of being generated by the o-dealkylation reaction. 35% of the metabolites of compounds TR442, TR421, TR428, TR390, and TR443 are generated by aromatic hydroxylation reaction, 25% of the metabolites of TR430, TR431, TR432, TR440, TR443 are generated by aliphatic hydroxylation reaction. Finally, 10% of the metabolites of TR432 and TR443 are generated by the dehydrogenation reaction.

### 6.4 Docking Molecular

For molecular docking, two docking results were generated using two scoring functions: the MolDock score and the PLANTS score. This method is validated through redocking, which verifies the RMSD values (Table **2**). In the MolDock score, the values varied between 0.154 Å and 1.684 Å. In the PLANTS score, they varied between 0.188 Å and 0.704 Å.

Then, the docking consensus was performed with both binding energy scores obtained: MolDock score and PLANTS score. It was observed in Table **SM7** (Supplementary Material) the results for targets AMPA, CaV, and GABAA and Table **SM8** (Supplementary Material) fp targets GAT-1 and NMDA. The TR430 molecule (N‐hexylnaphthalene‐2‐carboxamide) had the best consensus value among the 12 molecules and standard drugs for AMPA and GABAA targets. Thus, for AMPA, TR430 exhibited values of -260.184 kcal/mol. While the value of ethosuximide was -167.095 kcal/mol. And for GABAA the binding energy was -254.422 kcal/mol for TR430 and -230.579 kcal/mol for diazepam. For the NMDA target, the molecule that had the highest value was TR122 (-275.008 kcal/mol) compared to the other molecules and the standard drug, felbamate (-241.800 kcal/mol).

**Fig. (22):**
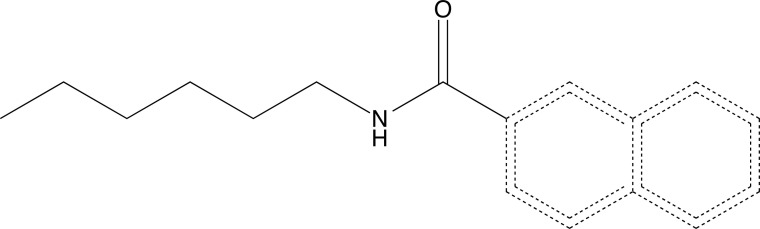
Chemical structure of compound TR430.

Finally, it was observed that TR117 presented a good consensus score for the CaV target and for the GAT-1 target. For CaV, the consensus score values were -199,900 kcal/mol and -104,872, respectively, for TR117 and ethosuximide. For GAT-1, the binding energy value of TR117 was -326,784 kcal/mol, and for tiagabine, it was -333,482 kcal/mol. Although TR117 had the best value among the other phenylpropanoids, the value of the drug control was superior to the molecule. Among all the observed interactions (Table **SM9**, **SM10**, **SM11**, **SM12**, and **SM13** - Supplementary Material), only the T430 (Fig. **22**) molecule was selected to evaluate the interactions based on best binding energy and multi-target activity.

The protein-ligand interactions presented by the TR430 with the AMPA target (Fig. **SM4** Supplementary Material) went through electrostatic pi-anion type interactions with the amino acid residue Glu193. It made hydrophobic pi-pi stacked-type interactions with Tyr61. In addition, van der Waals interactions with amino acids Leu191, Tyr190, Leu192, Leu138, Met196, Thr143, Thr174, Tyr220, Glu13, Pro89, Thr91, Leu90, and Arg96. The AMPA interactions with the drug ethosuximide exhibited conventional hydrogen bonding interactions with the amino acids Thr91, Arg96, and Pro89. Also performed hydrophobic pi-alkyl type interactions with Tyr61. And van der Waals interactions with amino acid residues Ser142, Leu90, Tyr220, and Glu193.

On the GABAA target, TR430 (Fig. **SM5**, Supplementary Material) showed a conventional hydrogen bond with the amino acid residue Arg67. It made two hydrophobic pi-pi stacked-type interactions with Tyr205 and three steric interactions with Thr202 (unfavorable interactions). In addition, it exhibited van der Waals interactions with the amino acids Arg173, Asp44, Leu128, Ser201, Arg120, Phe200, GLu155, Tyr97, Ser156, Tyr157, Gly158, Phe65, Leu118, Thr130, and Thr178. Diazepam interacted with the target through a covalent bond with the amino acid residue Arg67 and carbon-hydrogen-type hydrogen bonds with Thr130 (two interactions). In addition, it made hydrophobic pi-sigma type interactions with Phe46, pi-pi stacked type with Tyr205, and a pi-alkyl type with Phe65 (two interactions), Phe200 (two interactions), and Phe46 (one interaction). Finally, it showed van der Waals interactions with the amino acid residues: Ser201, Arg67, Thr202, Leu128, Leu118, Tyr157, Ser156, Gly158, Tyr97, Glu155, Val198, and Thr48.

## 7 DISCUSSION

A sample with 468 phenylpropanoids was used in this study. These secondary metabolites are abundantly distributed in the plant kingdom. They are present in different parts of plants: stem, leaf, flower, and others. Their occurrence differs between species and is influenced by the environment in which these plants are inserted [154, 155]. They also exhibit significant pharmacological activities such as antinociceptive, anti-inflammatory [156], anti-asthma [157], and anti-alzheimer [158].

The LBVS studies are important computational models of chemometrics for the analysis and development of drugs, which establish relationships between the biological activity of candidate molecules for possible drugs and their chemical structure; this relationship is made through numerical methods [159]. The LBVS models, in addition to training linear and non-linear models, perform the test with the database; this allows the prediction of valid results [160, 161].

LBVS analysis uses several statistical parameters to assess the quality of predictive models. One such parameter is the Matthews Correlation Coefficient (MCC), a statistical measure used to assess the predictive quality of classification models [162].

The MCC was calculated based on the true positive (TP), true negative (TN), false positive (FP), and false negative (FN) values. All of these values are considered to provide a balanced measure of the model's predictive ability. MCC values greater than 0.6 indicate that the model has better quality and reliability. Statistical parameters such as the Matthews coefficient can be used to assess efficacy and select the best predictive models for drug development [163].

Another metric that was used to evaluate the developed LBVS prediction model was the ROC curve. This curve was constructed by plotting true positive (TP) and false positive (FP) values for various model classification thresholds [164, 165]. The area under the ROC curve (AUC-ROC) was then calculated to provide a measure of the predictive power of the model. An AUC-ROC greater than 0.8 is considered a good quality model. This means that the model can properly discriminate between positive and negative samples. The higher the AUC-ROC, the better the model performance in terms of sensitivity and specificity [166]. In this way, the LBVS model has good predictive power and can be used to select molecules with the desired biological activity. This metric is particularly useful in screening large databases of compounds, allowing prioritization of compounds with the greatest potential for further investigation.

A study evaluated 700 articles that presented prediction models based on LBVS for targets against epilepsy and presented the importance of this methodology to evaluate molecules with drug potential [167]. In addition, another study corroborates the data presented, since they carried out a study of the LBVS in their work using a methodology similar to which was used in this work [168]. Thus, it can be stated that the 53 molecules of phenylpropanoids predicted to be active possibly have pharmacological activity against epilepsy. Then were evaluated their cytotoxic activity, oral absorption, bioavailability, and BBB permeability.

In the ADMET study, only 21 compounds among the 53 molecules evaluated showed promising partition coefficients (octanol/water), indicating that they possess lipophilic characteristics, suggesting possible permeability across membranes and cells. Due to this moderate lipophilicity, as demonstrated in the previously presented results, and in accordance with their moderate solubility, it can be expected that these compounds exhibit amphiphilic behaviour [169]. This means that they have a moderate affinity for both lipid substances and water. These compounds possess a molecular structure that contains polar (hydrophilic) and nonpolar (lipophilic) regions, allowing for moderate interaction with both substances [170-172].

This property can be advantageous in terms of bioavailability and permeability in biological systems, as the compound has a balanced tendency to dissolve in both aqueous and lipid environments. This can facilitate absorption, distribution, and interaction with biological targets [173].

The rate of oral absorption also indicates good expectations regarding the bioavailability of the studied molecules [174]. Bioavailability refers to the fraction of an administered drug that reaches the bloodstream intact and the speed at which this occurs. For a drug to be effective, it must be absorbed in sufficient quantity and within an appropriate timeframe [175].

When a drug is administered orally, it needs to be absorbed by the gastrointestinal tract before entering the bloodstream [176]. The rate of oral absorption is related to the speed at which the drug is absorbed and enters systemic circulation. A high absorption rate can result in greater bioavailability, as the drug is promptly and adequately absorbed. This allows the drug to quickly reach the site of action, producing the desired therapeutic effect [177].

When discussing the Lipinski rule regarding the studied compounds, which meet the criteria without any violations, we can highlight that these results indicate a high probability of good oral absorption and potential bioavailability [178]. This suggests that these compounds may have favorable characteristics for oral absorption. Corroborating the previously discussed data, where the oral absorption rate was excellent, as demonstrated in this study, along with moderate water solubility and a modeled partition coefficient, reinforces the expectation that these compounds can cross biological barriers and be efficiently absorbed by the gastrointestinal tract.

Furthermore, the 21 phenylpropanoid molecules showed a possible ability to cross the blood-brain barrier; this is due to the relationship between permeability and lipophilicity (LogP), molecular weight (MW), and TPSA [179]. Molecules that have a high lipophilicity and a low TPSA more easily cross the lipid barrier of the BBB [180]. In addition, a small molecular weight helps in the permeability of these molecules. Studies show that substances with a TPSA below 90 Å^2^ are more likely to reach the central nervous system [181].

The metabolism showed that 30% of the metabolites present in metabolism are generated through the O-dealkylation reaction. This reaction involves the removal of an alkyl group through the oxidation of the compound and is primarily catalyzed by certain hepatic enzymes. These enzymes remove the alkyl group and substitute it with hydrogen [182, 183].

According to the metabolic analyses conducted, it was found that approximately 35% might originate from aromatic hydroxylation, which involves the addition of a hydroxyl group to an aromatic ring within the structure. This reaction promotes the conversion of lipophilic substances into more water-soluble substances, facilitating their elimination by the body [184].

Another important point is aliphatic hydroxylation, which also increases the solubility of compounds by introducing a hydroxyl group into the aliphatic chain of the structure, making them more polar and enhancing excretion through bile or urine. According to the data, approximately 25% of the most abundant metabolites can originate from this reaction [185].

Dehydrogenation was also observed among the metabolites with the highest scores against CPY450. It is when the removal of a hydrogen atom occurs, resulting in unsaturation or an unsaturated group, making the molecule more reactive and possibly more water-soluble. According to the analyses, only compounds TR432 and TR443 showed potential metabolites originating from this reaction [186].

O-Dealkylation, Aromatic Hydroxylation, Aliphatic Hydroxylation, and Dehydrogenation are important reactions for the metabolism of a drug prototype. These metabolic processes can occur in the body, resulting in metabolites that may have different pharmacological properties, solubility, and half-life compared to the original drug. Additionally, these metabolic reactions play a crucial role in drug biotransformation, allowing the body to process and eliminate them more efficiently [187, 188].

To conclude the discussions on the ADMET properties, the compounds and their metabolites did not show toxicity risks in any of the parameters analyzed in this study (mutagenicity, carcinogenicity, toxic effects on the reproductive system, and irritability), supporting the previously discussed results that indicate promising pharmacokinetic potential.

Molecular docking is a technique used to evaluate based on the protein structure and with the possibility of predicting a possible mechanism of action for the studied molecules [189, 190]. One of the forms of evaluation is through the analysis of binding energy and target-molecule interactions. Both scores showed values lower than 2.0 Å for the RMSD; this parameter is used to validate the molecular docking and calculate the deviation of the atomic overlap. Therefore, the parameters evaluated in the study were significant and of quality since the crystalline molecule present in the protein was able to dock again with good alignment, which was expected in docking performance [191, 192].

Molecular docking consensus is a recent technique being used in bioinformatics [193]. It allows for correcting defects and possible errors that are present when only one molecular docking analysis is performed. In this way, it is possible to increase the number of algorithms or scoring functions to have more reliability and robustness of the obtained results [194, 195].

There are several ways to achieve consensus, but the significance of the method depends mainly on the performance of the algorithm being good [196]. In this way, we can state that the studies shown have significance and precision. The results showed values of the binding energy consensus greater than -143.757 kcal/mol. Therefore, the phenylpropanoids were able to connect with the binding site so that maximized affinity with the target. Among the set of molecules, TR430 and TR117 had the best values for binding energy.

Considering that epileptic seizures result from uncontrolled Glutamatergic and GABAergic pathways, consequently, GABAA and AMPA targets are closely related to the modulation of neuronal excitability [197, 198]. The molecule with the greatest therapeutic potential is TR430, as it was able to interact with both targets and could be a future multi-target molecule.

When analyzing the interactions made by TR430 with the AMPA target, it was possible to observe that the molecule behaved similarly to the control drug, ethosuximide, interacting with the same binding site. In addition, molecular docking analysis showed that in the evaluation of the antiepileptic activity of naphthalene-derived (arylalkyl)azoles, compound C1 (4-((1H-Imidazol-1-yl)methyl)-2-(naphthalen-2-yl)thiazole) interacted with the AMPA binding site with the residues Tyr61, Glu193, Glu13, Arg96, Val95, and Ala65 [199]. In another study that corroborates the findings of this research, the substance 6a ((Z)-N’-(1-methyl-2-oxoindolin-3-ylidene)-benzohydrazide) also interacted with the binding site through the amino acid residues Pro89, Glu193, Glu113, Leu138, Thr174, T y190, Met196, and Tyr16 [200].

Furthermore, *in vivo* and *in silico* studies on compounds derived from phthalazine-1,4-dione were conducted, and their antiepileptic activity was determined. Molecular docking showed that the molecules studied by the authors were able to interact with the binding site of the AMPA receptor through the same amino acid residues of TR430 [201].

The interactions at the binding site of GABAA with GABA consist of the amino acid residues Arg67, Phe65, Phe200, Thr202, Tyr157, and Tyr205 [146]. In the interaction of TR430 with this target, it was also observed that the molecule interacted with the same amino acid residues. Therefore, this substance has a possible activity similar to the endogenous agonist. Besides that, diazepam showed interactions with the same amino acids at the binding site.

At the GABAA receptor, the interaction of hydroxycitronellal was investigated to evaluate the antiepileptic activity. This study used another crystalline protein from PDB ID: 6HUJ, and the results corroborate the findings of TR430, in which the amino acids Arg67, Thr202, Tyr157, and Tyr205 are involved in the potentiation activity of the GABAA channel [202].

The TR430 activity may be partly due to its structural characteristics, the presence of naphthalene enables hydrophobic and electrostatic interactions, and the presence of the amide function enables hydrogen bonds with the binding sites.

There are few studies focused on the analysis of drugs with multi-target activity [203], the targets involved against epileptic seizures [204], and the antiepileptic activity of phenylpropanoid derivatives. TR430 is a promising substance with a possible antiepileptic activity. However, there is no substantial scientific evidence to support the antiepileptic activity of this compound beyond the results detailed in this study. Replication and further investigation are critical to establishing its effectiveness more comprehensively. The present results, although promising, need to be confirmed in follow-up studies.

## CONCLUSION

Thus, it is possible to conclude that there are few studies in the literature on the antiepileptic actions of phenylpropanoids and their derivatives. In the study carried out, the phenylpropanoids showed good results in the prediction models against epilepsy. The molecules that were selected in the virtual screening have possible good oral absorption good bioavailability, do not violate any Lipinski rule, and are able to cross the blood-brain barrier. In addition, neither the molecules nor their metabolites presented toxicity in the evaluated parameters. Molecular docking showed that two molecules presented the multi-target activity TR430 on AMPA and GABAA targets and TR117 on CaV and GAT-1 targets. However, the TR430 molecule exhibited good binding energy with both targets and their interactions. Therefore, TR430 is more likely to be a potential multi-target drug against epilepsy.

## Figures and Tables

**Table 1:** Data of possibly active molecules that are part of the applicability domain of the models.

**Model**	**Total**	**Domain**	**Activity Probability**
AMPA	8	Reliable	50.2% to 63.0%
CaV	5	Reliable	50.5% to 65.7%
GABAA	20	Reliable	50.4% to 66.1%
GAT-1	3	Reliable	50.2% to 58.1%
NMDA	20	Reliable	50.4% to 56.9%

**Table 2:** RMSD values for the analyzed targets.

**Targets**	**MolDock Score (Å)**	**Plants Score (Å)**
AMPA	0.245	0.396
CaV	0.997	0.704
GABAA	0.256	0.191
GAT-1	1.684	0.191
NMDA	0.154	0.188

## LIST OF ABBREVIATIONS

GABAB: γ-aminobutyric Acid Type B
GABAA-rho: γ-aminobutyric Acid Type A Subunits rho
IL-1β: *Interleukin*-1 beta
MMP3: Matrix Metalloproteinase-3
MMP13: Matrix Metalloproteinase-13
HPLC: High-performance Liquid Chromatography
MMFF94: Merck Molecular Force Field 94
AMBER: Assisted Model Building and Energy Refinement
SV2A: Synaptic Vesicle Glycoprotein 2A
SMILES: Simplified Molecular-input Line-entry System
