# Supplementary material for: Multi-target Phenylpropanoids Against Epilepsy
Source: Curr Neuropharmacol. 2024 May 27;22(13):2168–90. doi: 10.2174/1570159X22666240524160126 (PMC11337686; doi:10.2174/1570159X22666240524160126)
Supplement: Supplementary file 1 [file CN-22-2168_SD1.pdf]

Supplementary Material

Multi-target Phenylpropanoids Against Epilepsy

Teresa Carolliny Moreira Lustoza Rodrigues[2], Arthur Lins Dias[1], Aline Matilde Ferreira dos Santos[1], Alex France Messias Monteiro[2], Mayara Cecile Nascimento Oliveira[1], Hugo Fernandes Oliveira Pires[1], Natália Ferreira de Sousa[2], Mirian Graciela da Silva Stiebbe Salvadori[1], Marcus Tullius Scotti[2] and Luciana Scotti[2,3,*]

[1]Psychopharmacology Laboratory, Institute of Drugs and Medicines Research, Federal University of Paraíba, 58051-085, João Pessoa, Paraíba, Brazil; [2]Cheminformatics Laboratory, Institute of Drugs and Medicines Research, Federal University of Paraíba, 58051-900, João Pessoa, Paraíba, Brazil; [3]Teaching and Research Management, University Hospital Lauro Wanderley, Federal University of Paraíba, 58050-585, João Pessoa, PB, Brazil

Table SM1. Epilepsy disease target data found in ChEMBL.

Targets	ChEMBL ID	Type of Protein	Organism	Activity
GABAA	CHEMBL2095172	*Protein complex*	*Homo sapiens*	EC50
NMDA	CHEMBL1907603	*Protein complex*	*Homo sapiens*	IC50
NaV	CHEMBL4296	*Single protein*	*Homo sapiens*	IC50
GAT-1	CHEMBL1903	*Single protein*	*Homo sapiens*	IC50
KCNQ	CHEMBL2221348	*Protein complex*	*Homo sapiens*	EC50
CaV	CHEMBL1859	*Single protein*	*Homo sapiens*	EC50
GluR1	CHEMBL2009	*Single protein*	*Homo sapiens*	KI
GluR2	CHEMBL4016	*Single protein*	*Homo sapiens*	KI
GluR3	CHEMBL3190	*Single protein*	*Homo sapiens*	KI
GluR4	CHEMBL3595	*Single protein*	*Homo sapiens*	KI

1875-6190/24 $65.00+.00 **© 2024 Bentham Science Publishers**

Table SM2. Formulas used to calculate statistical parameters.

Statistical Parameters	Formulas
Precision	$\mathrm{PR} = \dfrac{\mathrm{VP}}{(\mathrm{VP} + \mathrm{FP})}$
Sensitivity	$\mathrm{SE} = \dfrac{\mathrm{VP}}{(\mathrm{VP} + \mathrm{FN})}$
Specificity	$\mathrm{SP} = \dfrac{\mathrm{VN}}{(\mathrm{VN} + \mathrm{FP})}$
Accuracy	$\mathrm{AC} = \dfrac{(\mathrm{VP} + \mathrm{VN})}{(\mathrm{VP} + \mathrm{VN} + \mathrm{FP} + \mathrm{FN})}$
Matthews Correlation Coefficient	$\mathrm{MCC} = \dfrac{(\mathrm{VP} + \mathrm{VN}) - (\mathrm{FP} + \mathrm{FN})}{\sqrt{(\mathrm{VP} + \mathrm{FP}) \times (\mathrm{VP} + \mathrm{FN}) \times (\mathrm{VN} + \mathrm{FP}) \times (\mathrm{VN} + \mathrm{FN})}}$
Activity Consensus	$\mathrm{ATV\ C} = \dfrac{((\mathrm{SP_D} \times \mathrm{ATV_D}) + (\mathrm{SP_C} \times \mathrm{ATV_C}) + (\mathrm{SP_V} \times \mathrm{ATV_V}) + (\mathrm{SP_R} \times \mathrm{ATV_R}))}{(\mathrm{SP_D} + \mathrm{SP_C} + \mathrm{SP_V} + \mathrm{SP_R})}$

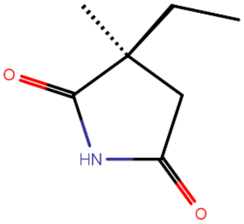

Ethosuximide

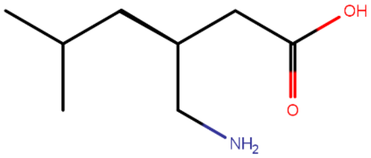

Pregabalin

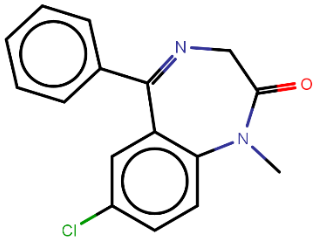

Diazepam

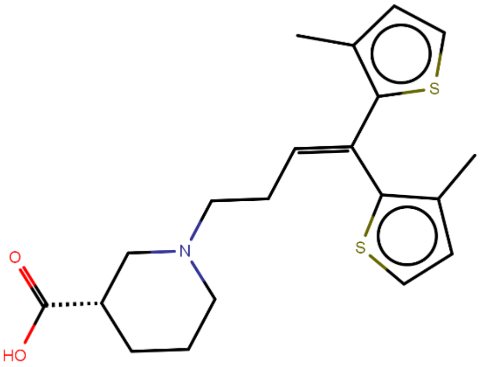

Tiagabine

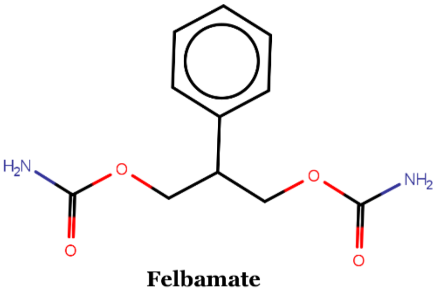

Felbamate

Fig. (SM1). 2D image of standard drugs used in molecular docking.

Table SM3. Values of statistical parameters obtained for all models.

Model	Validation	Precision	Sensitivity	Specificity	Accuracy	MCC
AMPA	Test	0.88	0.73	0.90	0.83	0.65
	Cross	0.81	0.88	0.78	0.83	0.67
CaV	Test	0.86	0.91	0.85	0.88	0.76
	Cross	0.73	0.91	0.75	0.82	0.65
GABAA	Test	0.83	0.77	0.83	0.80	0.61
	Cross	0.80	0.75	0.81	0.77	0.56
GAT-1	Test	0.87	0.90	0.87	0.88	0.77
	Cross	0.72	0.85	0.76	0.80	0.61
NaV	Test	0.86	0.87	0.79	0.84	0.66
	Cross	0.81	0.91	0.72	0.83	0.65
NMDA	Test	0.89	0.87	0.89	0.88	0.76
	Cross	0.66	0.92	0.63	0.76	0.56

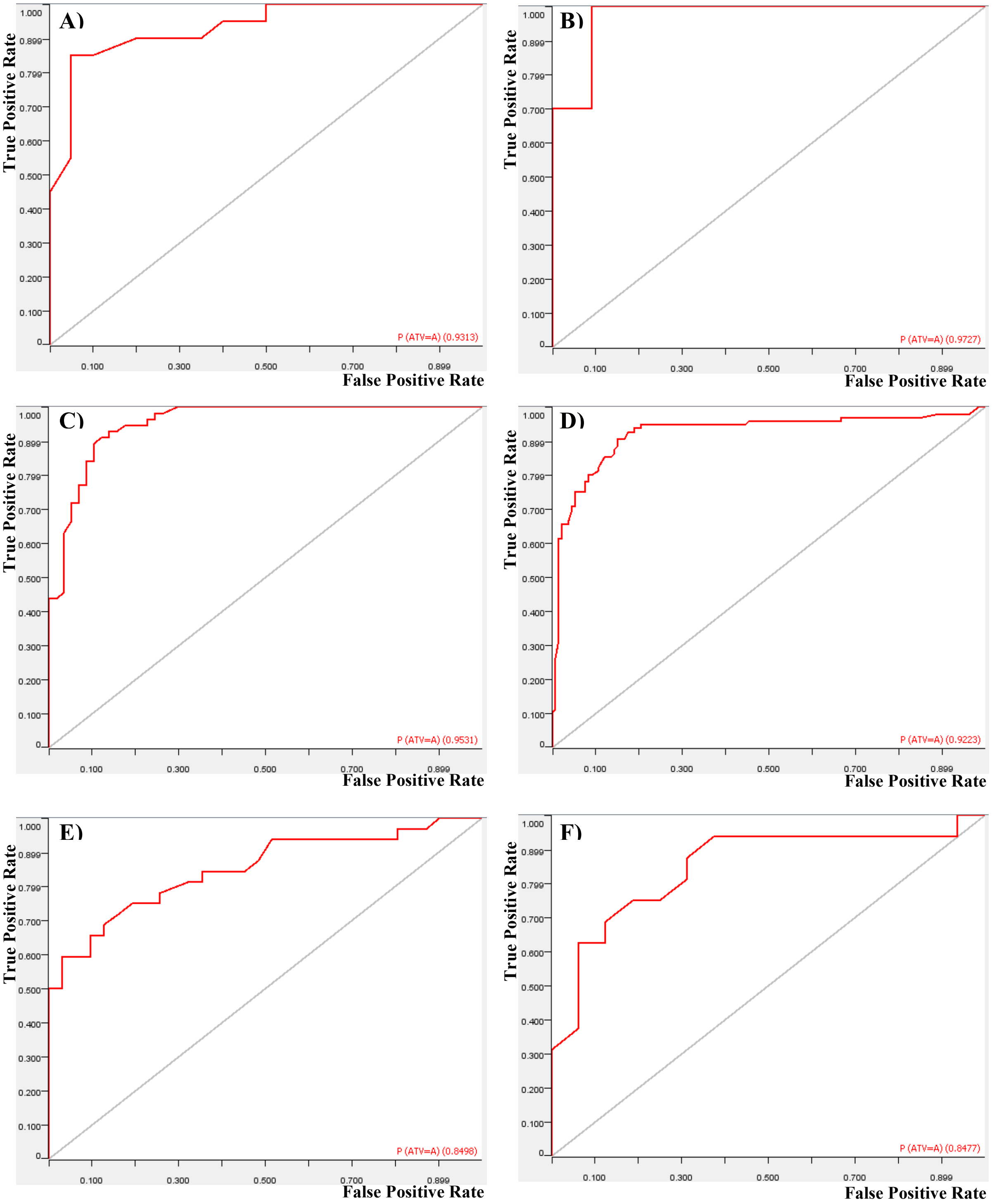

Fig. (SM2). ROC curves of the AMPA, CaV and GABAA models. A) AMPA test set; B) AMPA cross-validation; C) Cav test set; D) CaV cross-validation; E) GABAA test set; F) Cross-validation of GABAA. Legend: Red line = P(ATV=A) and gray line = random prediction.

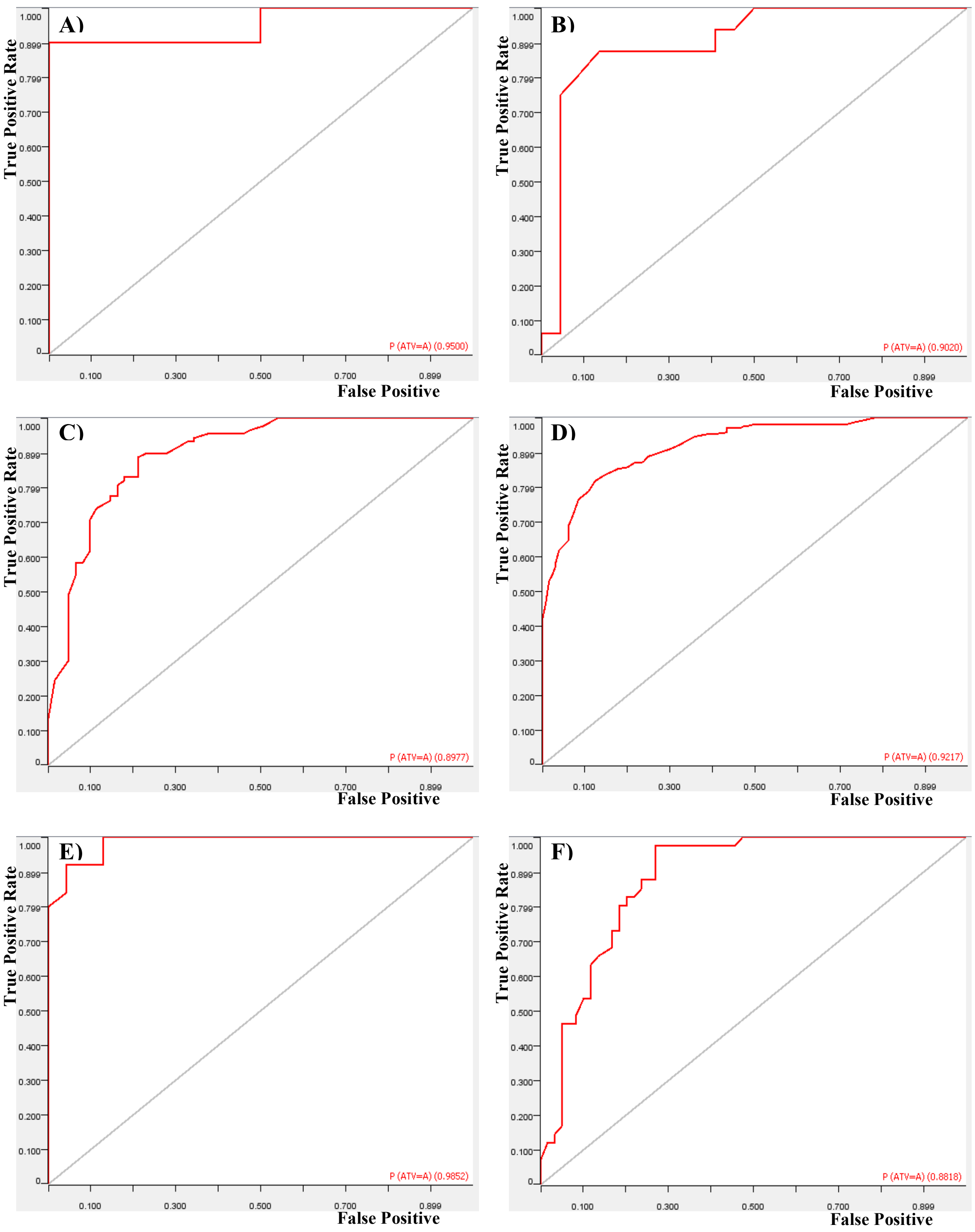

Fig. (SM3). ROC curves of GAT-1, NaV and NMDA models. A) Test set of GAT-1; B) GAT-1 cross-validation; C) NaV test set; D) NaV cross-validation; E) NMDA test set; F) NMDA cross-validation. Legend: Red line = P(ATV=A) and gray line = random prediction.

Table SM4. ADMET strengths calculated based on the molecular structures of each compound.

ID	MW	cLogP	cLogS	TPSA	%ABS	LIP	MUT	CAR	ESR	IRR	TOX
TR117	377.44	4.02	-4.26	56.79	89.47	0	No	No	No	No	No
TR122	330.38	4.07	-4.01	53.99	90.44	0	No	No	No	No	No
TR157	379.41	3.99	-5.48	66.02	86.29	0	No	No	No	No	No
TR421	261.32	3.71	-4.55	29.10	99.03	0	No	No	No	No	No
TR425	253.34	3.85	-4.80	29.10	99.03	0	No	No	No	No	No
TR426	239.32	3.51	-4.53	29.10	99.03	0	No	No	No	No	No
TR428	247.30	4.00	-4.73	29.10	99.03	0	No	No	No	No	No
TR430	255.36	4.52	-4.61	29.10	99.03	1	No	No	No	No	No
TR431	241.33	3.83	-4.23	29.10	99.03	0	No	No	No	No	No
TR432	227.31	3.37	-3.96	29.10	99.03	0	No	No	No	No	No
TR433	241.33	3.97	-4.45	29.10	99.03	0	No	No	No	No	No
TR436	275.35	4.00	-4.62	29.10	99.03	0	No	No	No	No	No
TR439	295.77	4.32	-5.29	29.10	99.03	1	No	No	No	No	No
TR440	241.33	4.06	-4.34	29.10	99.03	0	No	No	No	No	No
TR442	277.32	3.37	-4.25	49.33	92.05	0	No	No	No	No	No
TR443	303.40	4.90	-5.42	29.10	99.03	1	No	No	No	No	No
TR444	275.35	4.05	-4.89	29.10	99.03	0	No	No	No	No	No
TR448	254.33	4.32	-4.94	26.30	100.00	1	No	No	No	No	No
TR449	240.30	3.98	-4.67	26.30	100.00	0	No	No	No	No	No
TR456	242.32	4.44	-4.58	26.30	100.00	0	No	No	No	No	No
TR459	276.33	4.47	-4.76	26.30	100.00	1	No	No	No	No	No

MW = molecular weight (g/mol), TPSA = total polar topological surface area (Å^2), LIP = lipinski's rule, MUT= mutagenicity, CAR = carcinogenicity, ERS = toxic effect on the reproductive system, IRR = irritability and TOX = total toxicity.

Table SM5. Secondary metabolites of phenylpropanoids possibly originating in the liver.

ID	METID	Score	Smiles
TR157	Metabolite 28	100.00	COc1cc(C(Nc(cc2)ccc2Oc2ccccc2)=O)cc(OC)c1O
TR157	Metabolite 29	57.14	COc(cc(cc1O)C(Nc(cc2)ccc2Oc2ccccc2)=O)c1OC
TR117	Metabolite 40	100.00	COc1cc(C(NC(c2ccccc2)c2ccccc2)=O)cc(OC)c1O
TR117	Metabolite 41	84.07	COc(cc(cc1O)C(NC(c2ccccc2)c2ccccc2)=O)c1OC
TR122	Metabolite 50	100.00	CC(C)c(cc1)ccc1OC(c(cc1OC)cc(OC)c1O)=O
TR122	Metabolite 49	95.20	CC(C)c(cc1)ccc1OC(c(cc1O)cc(OC)c1OC)=O
TR442	Metabolite 55	100.00	Oc(ccc(CNC(c1cc2ccccc2cc1)=O)c1)c1O
TR442	Metabolite 60	93.89	Oc1ccc(CNC(c(ccc2c3)cc2ccc3O)=O)cc1
TR442	Metabolite 59	53.65	Oc1ccc(CNC(c2cc3cc(O)ccc3cc2)=O)cc1
TR421	Metabolite 74	100.00	Oc1ccc(cc(cc2)C(NCc3ccccc3)=O)c2c1
TR428	Metabolite 108	100.00	Oc(cc1)ccc1NC(c1cc2ccccc2cc1)=O
TR430	Metabolite 118	100.00	CC(CCCCNC(c1cc2ccccc2cc1)=O)O
TR431	Metabolite 135	100.00	CC(C)(CCNC(c1cc2ccccc2cc1)=O)O
TR432	Metabolite 150	100.00	CC(C)(CNC(c1cc2ccccc2cc1)=O)O
TR432	Metabolite 161	100.00	CC(C)=CNC(c1cc2ccccc2cc1)=O
TR439	Metabolite 195	100.00	Oc1ccc(cc(cc2)C(NCc(cc3)ccc3Cl)=O)c2c1
TR440	Metabolite 203	100.00	CC(CCCNC(c1cc2ccccc2cc1)=O)O
TR443	Metabolite 226	100.00	CC(C)c1ccc(CNC(c(ccc2c3)cc2ccc3O)=O)cc1
TR443	Metabolite 219	85.92	CC(C)(c1ccc(CNC(c2cc3ccccc3cc2)=O)cc1)O
TR443	Metabolite 232	85.92	CC(c1ccc(CNC(c2cc3ccccc3cc2)=O)cc1)=C

Table SM6. Possible reactions that gave rise to the hepatic metabolites with higher concentrations in the metabolic process.

ID	Reactions promoted by CP450			
	**O-Dealkylation**	**Aromatic Hydroxylation**	**Aliphatic Hydroxylation**	**Dehydrogenation**
TR157	Metabolite 28			
TR157	Metabolite 29			
TR117	Metabolite 40			
TR117	Metabolite 41			
TR122	Metabolite 49			
TR122	Metabolite 50			
TR442		Metabolite 55		
TR442		Metabolite 60		
TR442		Metabolite 59		
TR421		Metabolite 74		
TR428		Metabolite 108		
TR439		Metabolite 195		
TR443		Metabolite 226		
TR430			Metabolite 118	
TR431			Metabolite 135	
TR432			Metabolite 150	
TR440			Metabolite 203	
TR443			Metabolite 219	
TR432				Metabolite 161
TR443				Metabolite 232
Probability	30%	35%	25%	10%

Table SM7. Consensus ligand-receptor binding energies with AMPA, CaV, and GABAA targets.

Alvos	Moleculas	MolDock Score (kcal/mol)	PLANTS Score (kcal/mol)	Consenso Score (kcal/mol)
AMPA	TR157	-119.717	-386.534	-253.126
	TR117	-119.980	-380.137	-250.059
	TR122	-105.761	-405.405	-255.583
	TR442	-104.754	-404.148	-254.451
	TR421	-92.675	-401.665	-247.170
	TR428	-85.174	-396.344	-240.759
	TR430	-100.016	-420.352	-260.184
	TR431	-85.200	-413.384	-249.292
	TR432	-83.642	-406.544	-245.093
	TR439	-98.795	-402.335	-250.565
	TR440	-93.960	-417.483	-255.722
	TR443	-105.858	-403.748	-254.803
	Ethosuximide	-66.062	-268.127	-167.095
CaV	TR157	-58.208	-335.483	-196.846
	TR117	-52.840	-346.960	-199.900
	TR122	-51.331	-321.970	-186.650
	TR442	-40.650	-291.346	-165.998
	TR421	-37.968	-276.282	-157.125
	TR428	-32.387	-264.705	-148.546
	TR430	-45.171	-268.913	-157.042
	TR431	-36.580	-260.679	-148.630
	TR432	-38.167	-249.348	-143.757
	TR439	-40.231	-284.173	-162.202
	TR440	-39.635	-261.313	-150.474
	TR443	-49.109	-298.745	-173.927
	Pregabalin	-30.478	-208.420	-119.449
GABAA	TR157	-65.439	-357.237	-211.338
	TR117	-24.303	-316.752	-170.528
	TR122	-28.256	-397.773	-213.014
	TR442	-74.434	-400.370	-237.402
	TR421	-65.392	-400.277	-232.834
	TR428	-42.076	-391.810	-216.943
	TR430	-85.509	-423.336	-254.422
	TR431	-54.547	-412.195	-233.371
	TR432	-60.094	-414.857	-237.475
	TR439	-75.646	-401.740	-238.693
	TR440	-80.266	-420.059	-250.162
	TR443	-78.381	-403.141	-240.762
	Diazepam	-38.020	-423.137	-230.579

Table SM8. Consensus ligand-receptor binding energies with GAT-1 and NMDA targets.

Alvos	Moleculas	MolDock Score	PLANTS Score	Consenso Score
GAT-1	TR157	-98.117	-426.528	-262.323
	TR117	-107.092	-546.475	-326.784
	TR122	-101.621	-404.466	-253.044
	TR442	-90.013	-455.187	-272.600
	TR421	-87.038	-453.848	-270.443
	TR428	-74.384	-452.378	-263.381
	TR430	-92.337	-357.402	-224.870
	TR431	-88.712	-352.308	-220.510
	TR432	-73.217	-340.111	-206.664
	TR439	-85.049	-456.069	-270.559
	TR440	-88.843	-353.415	-221.129
	TR443	-93.964	-460.742	-277.353
	Tiagabine	-131.546	-535.418	-333.482
NMDA	TR157	-125.375	-409.535	-267.455
	TR117	-107.216	-367.197	-237.207
	TR122	-126.191	-423.824	-275.008
	TR442	-105.757	-400.940	-253.349
	TR421	-106.002	-436.454	-271.228
	TR428	-103.133	-402.211	-252.672
	TR430	-102.642	-399.146	-250.894
	TR431	-95.575	-393.140	-244.357
	TR432	-90.290	-390.535	-240.412
	TR439	-107.451	-430.153	-268.802
	TR440	-98.203	-395.244	-246.724
	TR443	120.277	-407.945	-143.834
	Felbamate	-110.502	-373.098	-241.800

Table SM9. Molecular interactions of phenylpropanoids with the AMPA target.

Molecule	Interactions	
	Types	Amino Acid Residues
TR157	Hydrogen	Arg96, Thr91, Tyr220
	Hydrophobic	Ala63, Val95, Tyr16, Pro89, Met196, Leu138
	Van der Waals	Arg64. Trp71, Gly53, Leu90, Ser142, Thr195, Glu13, Thr174, Gly141, Glu154
	Eletrostatic	Glu193
TR117	Hydrogen	Thr143, Thr174, Tyr190, Leu138, Ser140
	Hydrophobic	Leu192, Tyr61, Lys144
	Van der Waals	Thr195, Tyr16, Tyr220, Pro89, Arg96, Thr91, Leu90, Ser142, Gly141, Glu145, Thr137
	Steric	Leu191
	Eletrostatic	Glu13, Glu193
TR122	Hydrogen	Thr91, Leu90
	Hydrophobic	Leu138, Met196, Tyr16, Pro89
	Van der Waals	Thr195, Leu192, Tyr220, Thr174, Gly62, Gly141, Ser140, Ala63, Ser142, Thr143, Arg96
	Eletrostatic	Glu13, Glu193
TR442	Hydrogen	Gly62, Arg96
	Hydrophobic	Tyr61, Ala63
	Van der Waals	Lys60, Ser142, Gly141, Leu90, Thr91, Pro89, Tyr220, Tyr16, Met196, Thr195, Thr174
	Eletrostatic	Glu193, Glu13
TR421	Hydrogen	Arg96
	Hydrophobic	Tyr61, Ala63
	Van der Waals	Ser140, Gly141, Ser142, Thr174, Thr195, Met196, Tyr16, Tyr220, Pro89, Leu90, Thr91, Gly62, Lys60, Asn72
	Eletrostatic	Glu193, Glu13
TR428	Hydrogen	Arg96, Thr91
	Hydrophobic	Tyr61
	Van der Waals	Gly161, Ala63, Thr174, Met196, Tyr16, Tyr220, Pro89, Leu90, Ser142, Thr93
	Eletrostatic	Glu193, Glu13
TR430	Hydrophobic	Leu192, Met196, Tyr61
	Van der Waals	Leu191, Thr143, Tyr190, Leu138, Thr174, Tyr220, Glu13, Pro89, Thr91, Leu90, Arg96
	Eletrostatic	Glu193
TR431	Hydrophobic	Tyr190, Leu192, Leu138, Tyr61
	Van der Waals	Met196, Thr143, Leu191, Tyr220, Pro89, Leu90, Thr91, Arg96
	Steric	Thr174
	Eletrostatic	Glu193, Glu13

Molecule	Interactions	
	Types	Amino Acid Residues
TR432	Hydrogen	Thr195
	Hydrophobic	Met196, Pro15, Tyr16, Tyr61
	Van der Waals	Tyr199, Ser14, Trp255, Glu13, Thr195, Thr174, Leu138, Arg96, Leu90, Thr91, Pro89
	Eletrostatic	Glu193
TR439	Hydrogen	Arg96
	Hydrophobic	Ala63, Tyr61
	Van der Waals	Ser140, Gly141, Ser142, Thr174, Met186, Thr195, Tyr16, Tyr220, Pro89, Leu90, Thr91, Gly62, Lys60, Asn72
	Eletrostatic	Glu193, Glu13
TR440	Hydrophobic	Leu138, Met196, Leu192, Tyr61
	Van der Waals	Leu191, Tyr190, Arg,96, Leu90, Thr91, Pro89, Tyr220, Glu13, Thr174, Thr143
	Eletrostatic	Glu193
TR443	Hydrogen	Arg96
	Hydrophobic	Lys60, Ala63, Met196, Tyr61
	Van der Waals	Asn72, Gly62, Gly73, Gly141, Ser142, Thr91, Leu90, Pro89, Tyr220, Tyr16, Thr195, Thr174
	Eletrostatic	Glu13, Glu193
Ethosuximide	Hydrogen	Thr91, Arg96, Pro89
	Van der Waals	Ser142, Glu193, Tyr220, Leu90

Table SM10. Molecular interactions of phenylpropanoids with the CaV target.

Molecule	Interactions	
	Types	Amino Acid Residues
TR157	Hydrogen	Asn1764
	Hydrophobic	Phe1769, Met1767, Ile376, Phe369
	Van der Waals	Phe1731, Phe1773, Met1728, Leu1770, Phe372, Gly1766, Phe1765, Arg1763
TR117	Hydrogen	Asn1764
	Hydrophobic	Tyr370, Phe369
	Van der Waals	Leu1770, Phe372, Phe1769, Gly1766, Phe1765, Arg1763, Met279
	Eletrostatic	Met1767
TR122	Hydrogen	Asn1764
	Hydrophobic	Leu1770, Phe372, Met279, Phe369, Tyr370
	Van der Waals	Gly1766
	Eletrostatic	Met1767
TR442	Hydrogen	Gly1766
	Hydrophobic	Phe369, Phe1769
	Van der Waals	Phe1765, Arg1763, Asn1764, Leu1770, Phe372
	Eletrostatic	Met1767
TR421	Hydrogen	Gly1766
	Hydrophobic	Phe369, Phe1769
	Van der Waals	Phe372, Leu1770, Arg1763, Asn1764, Phe1765
	Eletrostatic	Met1767
TR428	Hydrogen	Gly1766
	Hydrophobic	Phe369, Leu1770
	Van der Waals	Phe372, Phe1769, Asn1764, Arg1763
	Eletrostatic	Met1767
TR430	Hydrophobic	Leu1770, Phe372, Phe369, Met1767
	Van der Waals	Arg1763, Asn1764, Gly1766, Phe1769
TR431	Hydrophobic	Phe1769, Leu1770, Phe372, Phe369
	Van der Waals	Arg1763, Gly1766, Asn1764
	Eletrostatic	Met1767
TR432	Hydrophobic	Phe1769, Leu1770
	Van der Waals	Phe369, Gly1766, Phe372, Asn1764, Arg1763
	Eletrostatic	Met1767
TR439	Hydrophobic	Phe1769, Phe369
	Van der Waals	Leu1770, Phe372, Arg1763, Asn1764, Phe1765, Gly1766
	Eletrostatic	Met1767
TR440	Hydrophobic	Leu1770, Phe372
	Van der Waals	Met279, Arg1763, Asn1764, Gly1766, Phe1769
	Eletrostatic	Met1767
TR443	Hydrophobic	Phe372, Phe1769, Phe369, Leu1770, Met279
	Van der Waals	Gly1766, Arg1763, Asn1764, Met1767, Tyr370
Pregabalin	Hydrophobic	Met1767, Phe369
	Van der Waals	Leu1770, Phe372, Gly1766, Tyr370, Arg1763, Asn1764

Table SM11. Molecular interactions of phenylpropanoids with the GABAA target.

Molecule	Interactions	
	Types	Amino Acid Residues
TR157	Hydrogen	Glu155, Ser156, Tyr205
	Hydrophobic	Leu128, Phe65, Phe200
	Van der Waals	Tyr97, Gly158, Arg120, Thr202, Ser201, Ser69, Gln68, Lys42, Thr43, Thr172, Arg173, Tyr129, Thr130, Leu99
	Steric	Tyr157, Leu118, Arg67, Asp44
	Covalent	Thr202
TR117	Hydrogen	Arg120, Leu118
	Hydrophobic	Tyr97, Tyr205, Phe65, Phe46, Phe200, Ser201, Leu128
	Van der Waals	Glu155, Ser156, Gly158, Tyr157, Thr130, Leu119, Tyr129, Thr172, Asp44, Thr48
	Steric	Arg67, Thr202
	Covalent	Leu128
TR122	Hydrogen	Tyr205, Ser156, Glu155, Arg67
	Hydrophobic	Arg120, Leu128, Thr202, Phe65, Leu99
	Van der Waals	Tyr129, Leu119, Gly158, Thr130
	Steric	Tyr97, Tyr157, Leu118
	Covalent	Leu128
TR442	Hydrogen	Arg67, Leu118
	Hydrophobic	Tyr157, Tyr205, Leu128
	Van der Waals	Arg120, Thr130, Phe65, Gly158, Ser156, Tyr97, Glu155, Phe200, Thr200, Ser201, Arg173, Thr172
	Covalent	Thr202
TR421	Hydrogen	Arg67
	Hydrophobic	Leu128, Tyr157, Tyr205
	Van der Waals	Thr172, Thr130, Leu118, Phe65, Gly158, Ser156, Tyr97, Glu155, Phe200, Arg120, Thr202, Ser201, Arg173
	Covalent	Thr202
TR428	Hydrogen	Arg,67
	Hydrophobic	Leu128, Tyr205, Phe65
	Van der Waals	Gln68, Tyr129, Thr130, Val198, Tyr97, Glu155, Ser156, Tyr157, Gly158, Leu118, Arg120, Ser201
	Steric	Thr202, Phe200
TR430	Hydrogen	Arg67
	Hydrophobic	Tyr205, Leu128
	Van der Waals	Thr172, Thr130, Leu118, Phe65, Gly158, Tyr157, Ser156, Tyr97, Glu155, Phe200, Arg120, Ser201, Asp44, Arg173
	Steric	Thr202
TR431	Hydrogen	Arg67
	Hydrophobic	Leu128, Tyr157, Tyr205

**Molecule**	**Interactions**	
	**Types**	**Amino Acid Residues**
	Van der Waals	Arg120, Thr130, Thr202, Phe65, Val189, Glu155, Tyr97, Ser156, Gly158, Leu118, Arg173, Ser201, Asp44
	Steric	Phe200
	Covalent	Thr202
TR432	Hydrogen	Arg67
	Hydrophobic	Leu118, Leu128, Tyr205
	Van der Waals	Tyr129, Phe65, Val198, Glu155, Tyr97, Ser156, Tyr157, Gly158, Arg120, Thr130
	Steric	Thr202, Phe200
TR439	Hydrogen	Arg67, Thr130
	Hydrophobic	Tyr157, Tyr205, Leu128
	Van der Waals	Thr172, Arg120, Leu118, Phe65, Gly158, Ser156, Tyr97, Glu155, Phe200, Ser201, Arg173, Thr172
	Steric	Thr202
	Eletrostatic	Arg67
TR440	Hydrogen	Arg67
	Hydrophobic	Leu128, Tyr205
	Van der Waals	Asp44, Ser69, Thr130, Leu118, Phe65, Tyr157, Gly158, Ser156, Tyr97, Glu155, Phe200, Arg120, Ser201, Thr172
	Steric	Thr202
TR443	Hydrogen	Arg67, Thr130
	Hydrophobic	Tyr157, Tyr205, Leu128
	Van der Waals	Asp44, Thr172, Thr173, Gly158, Ser201, Thr202, Leu118, Ser156, Glu155, Tyr97, Phe200, Phe65, Arg120, Asn55, Arg173
	Covalent	Thr202
Diazepam	Hydrogen	Thr130
	Hydrophobic	Phe46, Phe65, Phe200, Tyr205
	Van der Waals	Ser201, Arg67, Thr48, Val198, Glu155, Tyr97, Ser156, Gly158, Tyr157, Leu118, Leu128, Thr202
	Covalent	Arg67

Table SM12. Molecular interactions of phenylpropanoids with the GAT-1 target.

Molecule	Interactions	
	Types	Amino Acid Residues
TR157	Hydrogen	Leu303, Gly297, Ser295, Gly63, Gly65
	Hydrophobic	Ala305, Cys399, Tyr60, Leu300, Leu136, Phe294, Tyr140
	Van der Waals	Gly112, Ile304, Leu306, Tyr296, Ala61, Ile62, Asn66, Asn327, Leu392, Ser396, Gly403, Gly301
	Steric	Leu64
TR117	Hydrogen	Leu303, Ser295, Gly63, Thr400
	Hydrophobic	Tyr60, Ala305, Cys399, Tyr140, Leu136, Phe294
	Van der Waals	Leu306, Ser302, Phe98, Tyr296, Ala61, Gly297, Gly65, Asn66, Asn327, Leu64, Ser396, Leu300, Gln397
TR122	Hydrogen	Leu63, Leu64, Tyr140, Thr400, Gly297
	Hydrophobic	Leu306, Ala305, Cus399, Phe294
	Van der Waals	Ile304, Leu303, Gly307, Ala61, Asn327, Ser295, Asn66, Gly65, Leu136, Ser396, Gln397, Leu300, Tyr296, Ser302, Gly301
TR442	Hydrogen	Gly65, Thr400, Cys399, Leu303
	Hydrophobic	Tyr60, Leu306, Cys399, Ala305, Ser302
	Van der Waals	Asn66, Gly63, Ser295, Ala61, Ser396, Gly403, Ile304, Gly307, Gly297, Leu300, Leu136, Tyr140, Phe294, Leu64
TR421	Hydrogen	Thr400, Leu303
	Hydrophobic	Ala305, Leu306, Tyr60
	Van der Waals	Cys399, Ser396, Ala61, Ile62, Gly63, Ans327, Ser295, Gly65, Asn66, Leu64, Phe294, Tyr140, Leu136, Leu300, Ser302
TR428	Hydrogen	Leu303
	Hydrophobic	Tyr60, Ala305
	Van der Waals	Leu306, Ala61, Gly63, Asn66, Ser295, Gly65, Phe294, Leu64, Tyr140, Ser396, Leu136, Leu300, Thr400, Ser302
TR430	Hydrogen	Leu303
	Hydrophobic	Ala305, Leu306, Tyr60
	Van der Waals	Ile62, Asn66, Gly63, Phe294, Tyr140, Ser396, Thr400, Ser302, Ala61, Gly65, Asn327, Ser295, Leu64, Gly297, Leu300, Tyr296, Phe98
TR431	Hydrogen	Phe294, Ser295, Leu303
	Hydrophobic	Tyr140, Phe294, Tur60, Ser302, Ala305, Leu306
	Van der Waals	Leu64, Ser396, Gly297, Leu300, Cys399, Thr400, Gly65, Gly63, Asn66, Ala61, Tyr296, Ile62, Asn327, Phe98
TR432	Hydrogen	Ser295, Leu303
	Hydrophobic	Tyr60, Ala305, Leu306
	Van der Waals	Cys399, Thr400, Phe98, Phe294, Ser396, Leu300, Gly297, Leu136, Tyr140, Gly65, Leu64, Gly63, Ala61, Tyr296
TR439	Hydrogen	Leu303
	Hydrophobic	Tyr140, Leu136, Phe294, Tyr60, Leu306, Leu303, Ala305
	Van der Waals	Leu64, Ser295, Gly65, Gly63, Ser396, Cys399, Ala61, Tyr296, Ser302, Gly403, Ile304,

Molecule	Interactions	
	Types	Amino Acid Residues
		Thr400, Phe98, Leu300
TR440	Hydrogen	Leu303
	Hydrophobic	Tyr60, Ala305, Leu306, Cys399
	Van der Waals	Asn327, Asn66, Ile62, Gly63, Leu64, Gly65, Tyr140, Leu300, Ser396, Thr400, Ser302, Ile304, Gly307, Gly297, Phe284, Ser295
TR443	Hydrogen	Leu303
	Hydrophobic	Ala305, Cys399, Tyr60
	Van der Waals	Gly403, Ile304, Gly301, Gly112, Ser302, Thr400, Gly297, Leu300, Ser396, Leu136, Phe294, Leu64, Tyr140, Gly63, Ser295, Ala61, Asn327, Tyr296, Leu306, Gly403
Tiagabine	Hydrogen	Leu303, Gly63, Gly65
	Hydrophobic	Ala305, Leu306, Tyr60, Cys399, Phe294, Tyr140
	Van der Waals	Ile304, Gly403, Asn327, Ile62, Thr400, Ala61, Asn66, Ser295, Leu64, Leu136, Ser396, Leu300, Phe89, Gly297, Tyr296, Ser302

Table SM13. Molecular interactions of phenylpropanoids with the NMDA target.

Molecule	Interactions	
	Types	Amino Acid Residues
TR157	Hydrogen	Lys87, Asn171, Pro170, Asp215, Tyr214
	Hydrophobic	Ile136, Lys90, Lys87
	Van der Waals	Ala241, Gly135, Thr243, Thr134, Thr116, Thr174, Ser173, Ala216, His88, Gly172, Gly89, Arg121, Val169m Tyr245, Asn177
TR117	Hydrogen	Thr174, Asp215, Thr174, Ala241, Thr243, Thr116
	Hydrophobic	Tyr214, Ile136, His88
	Van der Waals	Leu115, Ser114, Arg232, Gly172, Val169, Thr168, Glu275, Asn118, Asn177, Glu273, Gly244, Gly135, Val218, Tyr245, Gly16, Leu115, Ser114
	Steric	Thr134
	Eletrostatic	Asp215
TR122	Hydrogen	Ser114, His88, Tyr214, Val169, Asp215
	Hydrophobic	Ala241, Ile136, His88
	Van der Waals	Glu273, Asn177, Ser173, Thr174, Glu175, Gly172, Lys87, Leu115, Thr116, Arg121, Tyr245, Ala216, Thr134, Gly135, Thr243, Ile117, Asn118
TR442	Hydrogen	Asp215, Ser173, Tyr214, Gly135
	Hydrophobic	Ala241, Ile136, Val169, Gly172
	Van der Waals	Glu273, Asn177, Tyr245, Gly175, Thr174, Thr168, Pro170, Lys87, His88, Ala216, Thr134, Thr243
TR421	Hydrogen	Tyr214, Asp215, Thr174
	Hydrophobic	Ile136, His88
	Van der Waals	Leu115, Ser114, Glu16, Val218, Tyr245, Gly172, Ala216, Thr134, Gly135, Thr243, Ala241, Asn177, Ser173, Thr116, Arg121
TR428	Hydrogen	Asp215, Thr174
	Hydrophobic	Ile136, His88, Ala241
	Van der Waals	Leu115, Ser114, Tyr214, Ser173, Asn177, Glu273, Thr243, Gly135, Thr134, Ala216, Gly172, Arg121
TR430	Hydrogen	Thr174, Gly172
	Hydrophobic	Ile136
	Van der Waals	Asn171, His88, Pro170, Val169, Lys87, Tyr214m Glu175, Ser173, Ala241, Gly135, Thr243, Thr134, Thr116, Asp215, Tyr245
TR431	Hydrogen	Thr174, Gly172
	Hydrophobic	Lys87, Ile136, Val169
	Van der Waals	His88, Glu175, Ser173, Tyr214, Gly135, Thr134, Thr243, Thr116, Tyr245, Asp215, Pro170, Asn171, Tyr214
TR432	Hydrogen	Thr174, Gly172
	Hydrophobic	His88, Lys87, Val169, Ile136
	Van der Waals	Glu175, Ser173, Ala241, Gly135, Thr134, Thr243, Thr116, Thr245, Asn171, Tyr214, Asp215, Pro170
TR439	Hydrogen	Tyr214, Arg121, Thr116, Thr174
	Hydrophobic	Leu115, His88, Ile136

Molecule	Interactions	
	**Types**	**Amino Acid Residues**
	Van der Waals	Glu16, Gly172, Tyr245, Ala216, Thr134, Gly135, Thr243, Ala241, Asn177, Ser173, Glu175, Ser114
TR440	Hydrogen	Thr174, Ser173
	Hydrophobic	Ile136
	Van der Waals	Pro170, Lys87, Val169, Tyr214, Glu175, Gly172, Ala241, Gly135, Thr243, Thr134, Tyr245, Thr116, Asp215, His88, Asn171
TR443	Hydrogen	Gly135, Tyr214
	Hydrophobic	Ile136, Ala241
	Van der Waals	Glu119, Asn118, Thr243, Ile117, Thr116, Thr134, Ala216, Ser173, Asp215, Thr174, Glu175, Gly172, Val169, Pro170, Lys87, His88, Tyr245, Ser137, Asn177, His272, Thr242, Glu273
Felbamate	Hydrogen	Thr174, Gly172, His88, Ser114, Arg121, Thr116
	Hydrophobic	Ile136
	Van der Waals	Val169, Glu175, Tyr214, Leu115, Tyr245, Thr134, Ala216, Gly135, Thr243, Ser173

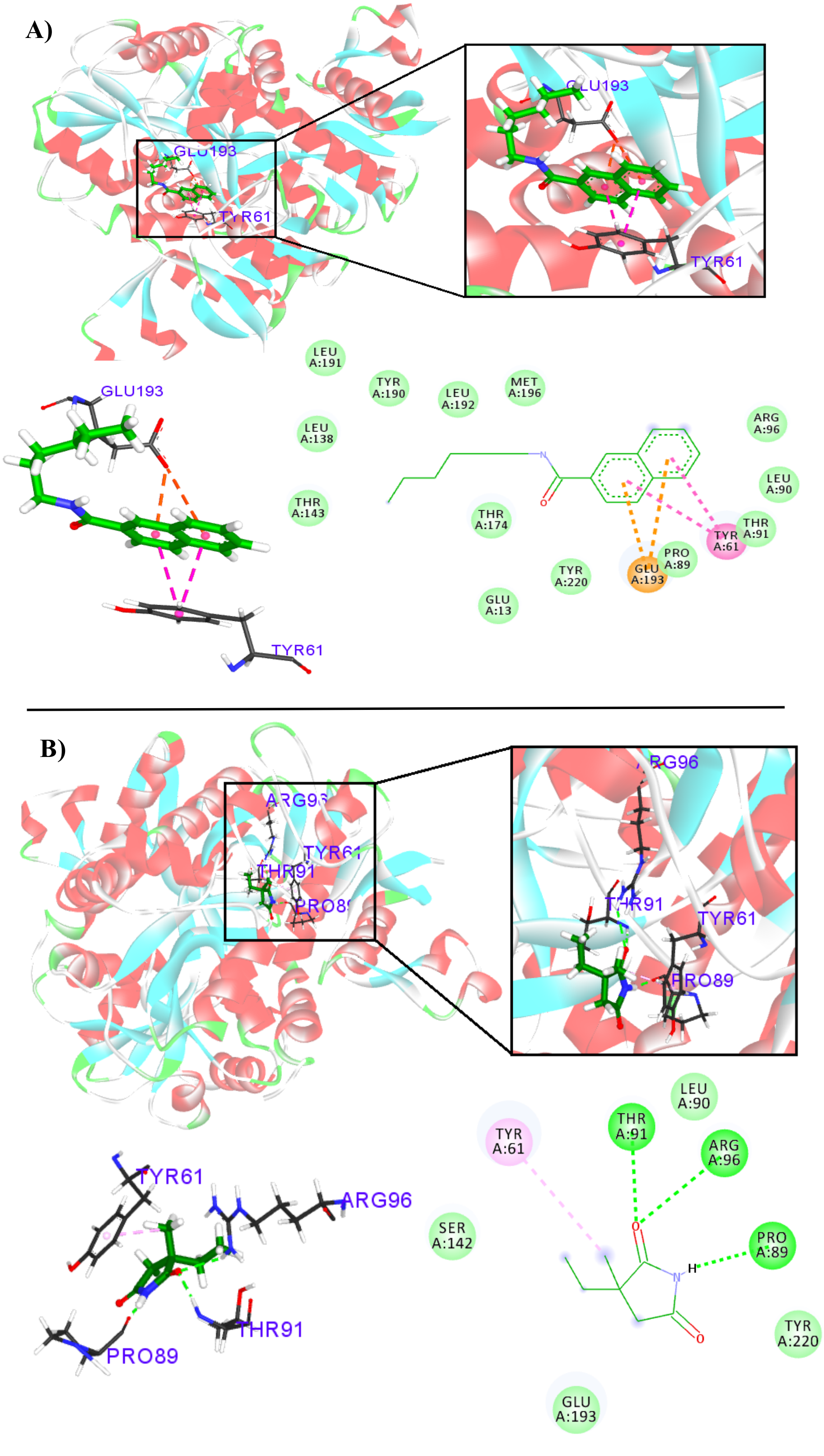

Fig. (SM4). 3D and 2D interactions between AMPA protein with A) TR430 phenylpropanoid and B) ethoxusimide. Hydrogen bonds are highlighted in green with dotted lines; hydrophobic interactions are highlighted in pink, steric interactions are highlighted in orange and van der Waals interactions are highlighted in green without the dotted line.

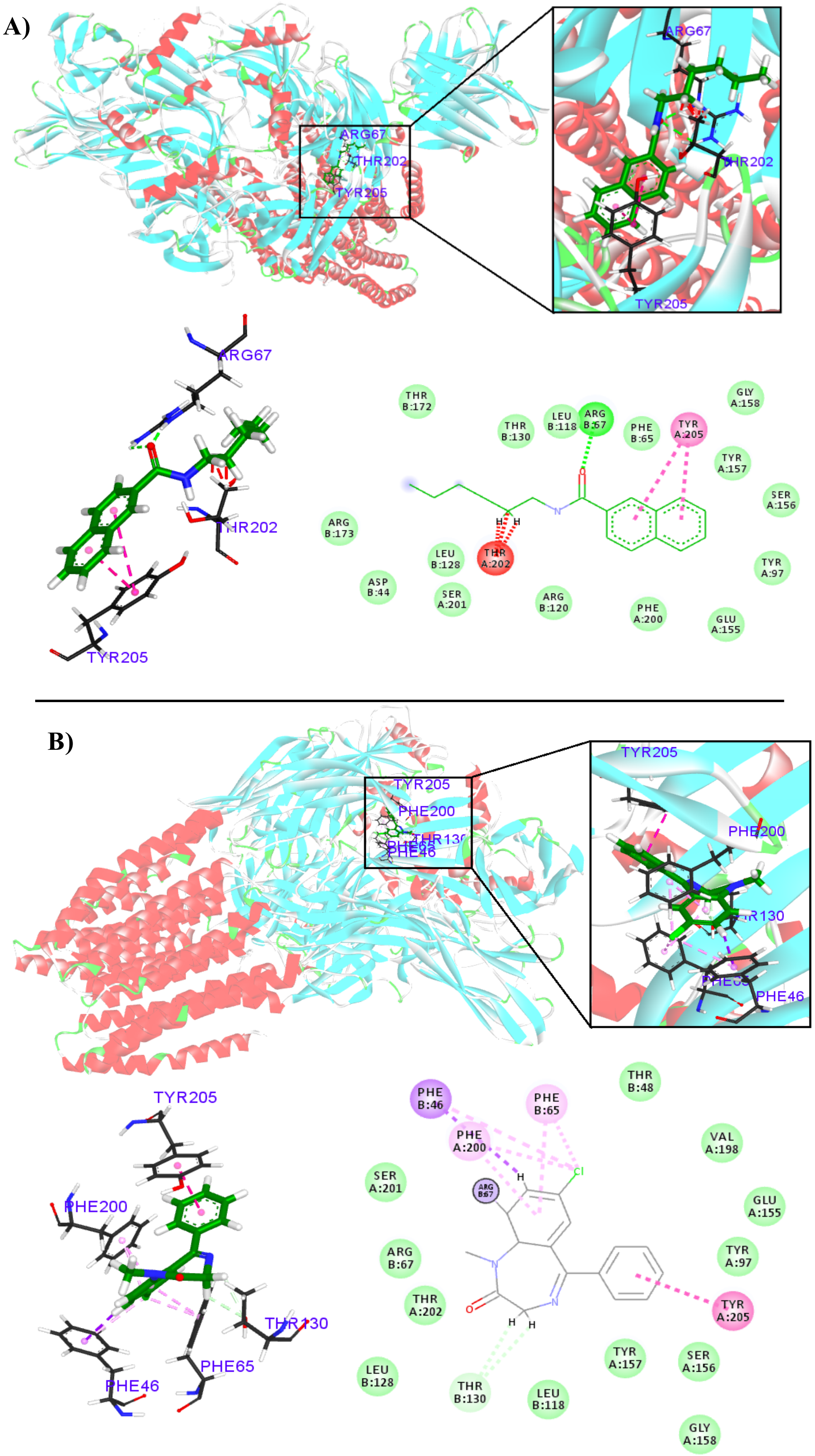

Fig. (SM5). 3D and 2D interactions between GABAA protein with A) TR430 phenylpropanoid and B) diazepam. Hydrogen bonds are highlighted in green with dotted lines; covalent bond highlighted in purple connected to the molecule, hydrophobic interactions are highlighted in pink and purple with dotted lines and van der Waals interactions are highlighted in green without the dotted line.
